# Epigenetic drugs in cancer therapy: mechanisms, immune modulation, and therapeutic applications

**DOI:** 10.1186/s43556-025-00373-5

**Published:** 2025-12-03

**Authors:** Chen Ma, Junkai Cheng, Jian Gu, Qin Wang

**Affiliations:** 1 College of Pharmacy and Food, Southwest Minzu University, Chengdu, 610225 Sichuan China; 2 Department of Pathology, Yong Loo Lin School of Medicine, National University of Singapore, Singapore, 117597 Singapore Singapore

**Keywords:** Epigenetic regulation, DNA methylation, Histone modifications, Tumor immunity, Clinical translation

## Abstract

Epigenetic regulation is a fundamental mechanism controlling gene expression and cellular function, primarily mediated through reversible modifications such as DNA methylation, histone acetylation, and chromatin remodeling. Dysregulation of critical epigenetic enzymes, including histone deacetylases (HDACs), DNA methyltransferases (DNMTs), and bromodomain and extraterminal domain (BET) proteins, has been closely associated with tumor initiation, progression, metastasis, immune evasion, and resistance to conventional therapies. Targeting these epigenetic regulators with small-molecule inhibitors or degraders has emerged as a promising therapeutic strategy, capable of reprogramming aberrant transcriptional networks and reshaping the tumor microenvironment. Beyond direct cytotoxic effects, epigenetic drugs have demonstrated the ability to enhance antitumor immunity by restoring antigen presentation, promoting immunogenic cell death, modulating cytokine profiles, and reversing local immune suppression. Recent preclinical and clinical studies have highlighted the potential of combining epigenetic therapies with immune checkpoint inhibitors to achieve synergistic antitumor responses and overcome resistance mechanisms. This review provides a comprehensive summary of the mechanisms of action, pharmacological characteristics, and clinical applications of epigenetic drugs, with a focus on innovative combination strategies and ongoing translational advancements. We also discuss future directions, emphasizing the need to improve drug specificity, minimize off-target effects, integrate personalized immunotherapeutic approaches, and identify predictive biomarkers to optimize patient selection and clinical outcomes. Overall, epigenetic therapy represents a versatile and evolving avenue for precision oncology with broad implications for tumor control and immunomodulation.

## Introduction

**Fig. 1:**
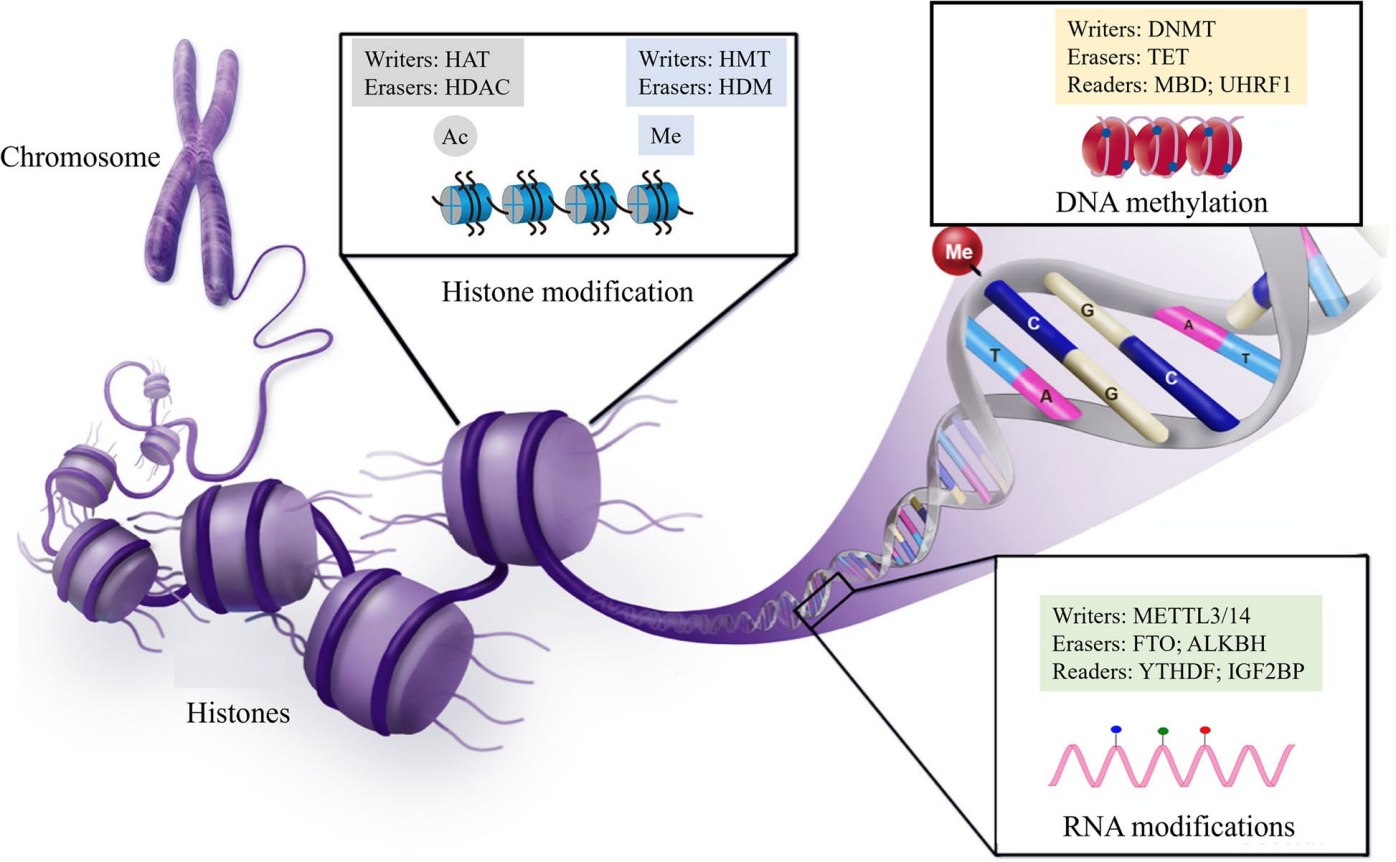
Epigenetic modifications are dynamic and reversible processes regulated by functionally complementary enzymes, making them promising targets for disease therapeutics. Histone acetyltransferase (HAT) and deacetylase (HDAC) add and remove acetyl groups, while histone methyltransferase (HMT) and demethylase (HDM) modulate histone methylation. DNA methylation is catalyzed by DNMT and reversed by TET proteins, with MBDs and UHRF1 recognizing methylated DNA. RNA N6-methyladenosine (m6A) is deposited by METTL3/14, removed by FTO and ALKBH proteins, and interpreted by reader proteins such as YTHDF and IGF2BP, collectively regulating gene expression

Epigenetic drugs have emerged as a powerful class of therapeutic agents in modern oncology, offering precise modulation of gene expression without altering the underlying DNA sequence [1]. These drugs target key epigenetic enzymes involved in DNA methylation, histone modification, and chromatin remodeling, thereby reversing aberrant transcriptional programs that drive tumorigenesis and immune evasion. Unlike permanent genetic mutations, epigenetic alterations are dynamic and reversible, making them particularly attractive for therapeutic intervention [2–4]. Epigenetic modifications are orchestrated by three main classes of regulatory proteins: writers, erasers, and readers, as shown in Fig. 1 [5]. Writers, such as DNA methyltransferases (DNMTs) and histone acetyltransferases (HATs), install chemical marks that activate or silence gene transcription; erasers, including histone deacetylases (HDACs) and ten-eleven translocation (TET) enzymes, remove these marks; and readers, such as bromodomain and extra-terminal (BET) proteins and methyl-CpG-binding proteins (MBPs), recognize these modifications to recruit transcriptional machinery [6, 7]. Dysregulation of these regulators drives tumor initiation, progression, metastasis, and therapeutic resistance, while also profoundly reshaping inflammatory and immune signaling within the tumor microenvironment. Specifically, inhibition of aberrant epigenetic regulators can suppress transcriptional programs driven by pro-inflammatory stimuli such as NF-κB and STAT3, which induce IL-6 and TNF-α expression. For example, HDAC inhibitors downregulate pro-inflammatory cytokines while enhancing the production of the anti-inflammatory cytokine IL-10, exerting both anti-inflammatory and antitumor effects. Inhibition of histone or DNA methyltransferases can restore the expression of silenced immune-related genes, including those involved in antigen presentation and interferon signaling [8–11].

Although considerable progress has been made in the development of epigenetic drugs, the rapidly expanding landscape of epigenetic regulators, novel drug modalities, and immune-modulatory mechanisms necessitates a comprehensive and updated synthesis. Emerging evidence demonstrates that epigenetic drugs not only exert tumor-intrinsic anticancer effects but also modulate antitumor immunity by restoring antigen presentation, reactivating silenced tumor-associated antigens, regulating cytokine production, and reversing T cell exhaustion [12–16]. For instance, aberrant HDAC and DNMT activity can downregulate MHC class I expression and promote T cell dysfunction, while BET proteins contribute to immune suppression through transcriptional activation of checkpoint and inflammatory genes. Innovative strategies, including metabolic modulators such as IDH1/2 inhibitors, locus-specific CRISPR-dCas9 systems, proteolysis-targeting chimeras (PROTACs), and metal-based agents, offer enhanced specificity, efficacy, and combinatorial potential. This review is therefore intended to provide an integrated perspective that captures mechanistic insights, immunological impact, and translational relevance of current and emerging epigenetic therapies.

Here, we provide a comprehensive overview of epigenetic drugs targeting HDACs, DNMTs, and BET proteins, emphasizing their immunomodulatory mechanisms and therapeutic applications in cancer. We highlight metabolic modulators, emerging regulators, and innovative technologies that enable precise epigenetic control. Novel therapeutic strategies, including PROTACs and metal-based agents, are discussed for their enhanced potency and specificity. We further summarize ongoing clinical progress, challenges such as off-target effects and resistance, and propose strategies for integrating epigenetic therapies into personalized cancer immunotherapy through rational combinations and biomarker-guided patient selection. This structured approach provides a clear, sequential exploration from mechanistic foundations to translational and clinical applications, offering a holistic perspective on the evolving landscape of epigenetic drug development.

## Classification and mechanisms of epigenetic drugs

Epigenetic modifications play a crucial role in gene regulation, and their dysregulation is a hallmark of cancer. One of the earliest discoveries in this field revealed that DNMT inhibitors can effectively induce DNA demethylation, leading to the reactivation of epigenetically silenced genes [6]. This breakthrough laid the foundation for targeting epigenetic changes in cancer therapy [17]. Over the past few decades, research has uncovered the complexity of epigenetic regulation, which extends beyond DNA methylation to include covalent histone modifications, chromosome remodeling, and RNA modification [8, 18]. Therefore, A growing number of these agents, such as DNMT inhibitors (e.g., azacitidine), HDAC inhibitors (e.g., vorinostat), and EZH2 inhibitors (e.g., tazemetostat), have received regulatory approval from the Food and Drug Administration (FDA) and other international agencies for the treatment of hematologic malignancies and solid tumors, reflecting the therapeutic promise of this class of drugs, as summarized in Table 1.

**Table 1:** FDA-Approved Epigenetic-Regulatory Drugs for Cancer Therapy (2004–2025)

Drugs	Comercial Name	Company	Category	Indications	Year Approved
Azacitidine	Vidaza	Celgene	DNMTi	AML, CMML, MDS	2004
Decitabine	Dacogen	Otsuka	DNMTi	AML, CMML, MDS	2006
Vorinostat	Zolinza	Merck	Pan-HDACi	Cutaneous T‑cell lymphoma (CTCL)	2006
Romidepsin	Istodax	Celgene	Class I HDACi	CTCL, peripheral T‑cell lymphoma (PTCL)	2009
Belinostat	Beleodaq	Spectrum	Pan-HDACi	Relapsed/refractory PTCL	2014
Panobinostat	Farydak	Novartis	Pan-HDACi	Multiple myeloma	2015
Enasidenib	Idhifa	Celgene	IDH2i	Relapsed/refractory AML (IDH2 mutation)	2017
Ivosidenib	Tibsovo	Servier	IDH1i	Relapsed/refractory AML (IDH1 mutation)	2018
Tazemetostat	Tazverik	Epizyme	EZH2i	Epithelioid sarcoma, relapsed follicular lymphoma	2020
Olutasidenib	Rezlidhia	Forma Therapeutics	IDH1i	Relapsed/refractory AML (IDH1 mutation)	2022
Vorasidenib	Voranigo	Servier	IDH1/IDH2i	Grade 2 astrocytoma or oligodendroglioma (IDH1/IDH2 mutation)	2024
Revumenib	Revuforj	Syndax	Menin-i	Relapsed/refractory KMT2A‑rearranged acute leukemia	2024

### DNA methyltransferase inhibitors

DNA methylation is a heritable epigenetic mark that plays a central role in transcriptional repression, especially when enriched at gene promoters. DNMTs are key epigenetic regulators that catalyze the transfer of a methyl group from S-adenosylmethionine (SAM) to the 5-position of cytosine residues in CpG dinucleotides, leading to gene silencing. Although DNMT1, DNMT3a, and DNMT3b have a similar structural organization, with an N-terminal regulatory domain and a C-terminal catalytic domain, these enzymes exhibit distinct functions and expression patterns [16]. DNMT1, the primary maintenance methyltransferase, plays a dual role in preserving pre-existing DNA methylation patterns during DNA replication and cell division, while also contributing to DNA methylation repair. In contrast, DNMT3a and DNMT3b, classified as de novo methyltransferases, establish new methylation marks on previously unmethylated DNA sequences, thereby shaping novel epigenetic landscapes [15]. The recruitment of DNMT3a and DNMT3b to specific DNA regions is guided by transcription factors (TFs) such as CTCF, Sp1, YY1, NRSF/REST, FOXA1, and SALL4 [13, 14]. Additionally, DNMT2 and DNMT3L are structurally related to the DNMT family but lack cytosine methyltransferase activity. DNMT3L enhances the enzymatic function of DNMT3a and DNMT3b by increasing their affinity for SAM. Meanwhile, DNMT2 plays a distinct role in RNA methylation, targeting non-coding RNAs (ncRNAs) such as transfer RNA (tRNA), ribosomal RNA (rRNA), and nuclear RNA (nRNA), thereby influencing post-transcriptional regulation. DNA methylation is fundamental to various cellular processes, including embryonic development, X-chromosome inactivation, and genomic imprinting [12, 16]. However, dysregulated DNA methylation patterns, characterized by hypermethylation of tumor suppressor genes and global DNA hypomethylation, are widely recognized as hallmarks of cancer. These aberrant epigenetic modifications contribute to genomic instability, aberrant gene expression, and tumor progression.

DNA methyltransferase inhibitors (DNMTi) have gained significant attention as promising therapeutic agents in epigenetic cancer therapy, offering potential for targeted reprogramming of aberrant DNA methylation landscapes. DNMTis represent a foundational class of epigenetic therapeutics, particularly effective in reversing aberrant DNA methylation patterns that silence tumor suppressor genes, immune-related genes, and differentiation pathways in cancer. These inhibitors are generally divided into nucleoside analogs, non-nucleoside small molecules, and natural compounds-derived DNMTis, each characterized by distinct mechanisms of action and pharmacologic profiles (Table 2). In addition to gene reactivation, DNMTi also regulates key intracellular signaling pathways for cell survival, cell extinction, and immune response. In the action network of DNMTis, the regulation of the PI3K/Akt pathway is an important branch. Abnormal activation of this pathway is a core feature of most tumors, and DNMTis can achieve intervention by restoring the expression of negative regulatory factors in the pathway. For instance, the tumor suppressor gene phosphatase and tensin homolog gene (PTEN) is a key inhibitor of the PI3K/Akt pathway. Gravina et al. found that in some tumors, PTEN is often epigenetically silenced due to promoter methylation, while DNMTi can reactivate PTEN through demethylation. This further downregulates the phosphorylation level of Akt and weakens the cell survival signal [19]. In addition, DNMT1 itself is directly related to the activation of the PI3K/Akt pathway. Liu et al. confirmed that the transcription factor ZNF191 can directly bind to the DNMT1 promoter and upregulate its expression. The high expression of DNMT1 will enhance the phosphorylation of Akt in hepatoma cells and activate the PI3K-Akt pathway to promote tumor growth. Conversely, knocking down DNMT1 will significantly weaken the activation of this pathway [20]. These two studies have revealed the dual mechanisms of DNMTis' intervention in the PI3K/Akt pathway from two dimensions: "reactivation of pathway inhibitors" and "regulation of key pathway molecules".

**Table 2:** Overview of DNMT inhibitors in cancer therapy

Category	Drugs	Mechanism of Action	Key Features	NCT identifier
Nucleoside Analog Inhibitors	Azacytidine	Incorporated into DNA and RNA; covalently binds DNMTs to inhibit methylation	Replication-dependent; induces global DNA hypomethylation; cytotoxic to proliferating cells	NCT00384839 (Phase 2, Completed), NCT00996515 (Phase 1, Completed), NCT00382590 (Phase 2, Completed), etc
	Decitabine	DNA incorporation; traps DNMTs and blocks methyltransferase activity	Replication-dependent; effective in hematologic malignancies; dose-limiting myelosuppression	NCT02076191 (Phase 2, Completed), NCT02257138 (Phase 2, Completed), NCT05717621 (Phase 1, Completed), etc
	Guadecitabine	Prodrug of decitabine; incorporated into DNA to inhibit DNMTs	Extended half-life; sustained demethylation; potential in solid tumors	NCT03075826 (Phase 2, Completed), NCT01896856 (Phase 2, Completed), NCT02429466 (Phase 1, Completed), etc
	Zebularine	DNA incorporation; covalent DNMT inhibition	More stable and less toxic than azacytidine; primarily used in preclinical models	Preclinical
Non-Nucleoside Inhibitors	RG108	Directly binds DNMT catalytic site without DNA incorporation	Replication-independent; reversible inhibition; lower cytotoxicity	Preclinical
	SGI-1027	Inhibits DNMT activity by blocking cofactor binding	Selective for DNMT1; minimal DNA incorporation; reversible	Preclinical
	GSK3685032	Allosteric DNMT1 inhibitor	Replication-independent; improved selectivity; potential combination with immunotherapy	Preclinical
	MC3343	Selective DNMT1 inhibition; binds catalytic domain	Reversible; less toxic; may synergize with other epigenetic agents	Preclinical
	CM-272	Dual DNMT1 and G9a inhibition	Multi-target approach; affects DNA methylation and histone methylation	Preclinical
Natural Compounds	Curcumin	Partial DNMT inhibition; modulates gene expression via multiple pathways	Anti-inflammatory; antioxidant; low toxicity; dietary polyphenol	NCT01333917 (Phase 1, Completed), NCT01201694 (Phase 1, Completed), NCT02017353 (Phase 2, Completed), etc
	EGCG	Inhibits DNMT activity; interacts with methyl donors	Antioxidant; epigenetic modulation; low systemic toxicity	NCT06924749 (Phase 2, Completed), NCT05039983 (Phase 1, Completed), NCT04177693 (Phase 1, Completed), etc
	Quercetin	Modulates DNMT function; affects methylation of tumor suppressor genes	Low toxicity; multi-pathway effects	NCT03476330 (Phase 2, Active, not recruiting), NCT01912820 (Phase 1, Completed), NCT06355037 (Phase 2, Recruiting), etc
	Genistein	Inhibits DNMTs; modulates estrogen-related gene methylation	Potential chemopreventive activity	NCT01985763 (Phase 2, Completed), NCT00244933 (Phase 2, Completed), NCT00118040 (Phase 2, Completed), etc
	Resveratrol	Epigenetic modulation via DNMT inhibition and SIRT1 activation	Multiple signaling pathways; antioxidant	NCT00256334 (Phase 1, Completed), NCT00920803 (Phase 1, Completed), NCT00098969 (Phase 1, Completed), etc
	Sulforaphane	DNMT inhibition; induces demethylation of tumor suppressor genes	Low toxicity; epigenetic chemopreventive effects	NCT01228084 (Phase 2, Completed), NCT03232138 (Phase 2, Completed), NCT00946309 (Phase 2, Completed), etc
	Apigenin	Partial DNMT inhibition; regulates transcriptional activity	Anti-inflammatory; dietary compound	Preclinical

#### Nucleoside analog DNMTis

Nucleoside analog DNMTis, such as Azacytidine and Decitabine, are incorporated into DNA or RNA during the S-phase of the cell cycle [21]. Once incorporated, they trap DNMT enzymes in covalent adducts, leading to enzyme degradation and passive DNA demethylation during subsequent cell divisions. Azacitidine incorporates into both DNA and RNA, exerting a dual function by disrupting DNA methylation and protein translation, while Decitabine acts more selectively on DNA, leading to more focused DNMT inhibition. Both are FDA-approved for the treatment of myelodysplastic syndromes (MDS) and acute myeloid leukemia (AML), and remain the cornerstone of clinical epigenetic therapy. However, these compounds are limited by short plasma half-life, cytotoxicity to non-cancerous cells, and dependence on DNA replication, restricting their efficacy in slow-proliferating tumors. To address these limitations, next-generation nucleoside analogs have been developed. One such agent is Guadecitabine, a dinucleotide prodrug of decitabine that is resistant to deamination by cytidine deaminase, resulting in prolonged in vivo stability and enhanced efficacy in both hematologic and solid tumors [22]. Other notable candidates include Zebularine, a cytidine analog with oral bioavailability and lower toxicity, and S110, a second-generation azacytidine prodrug with improved pharmacokinetics [23].

Clinically, several pivotal trials have confirmed that nucleoside analog DNMT inhibitors (DNMTis) can translate epigenetic reprogramming into meaningful survival benefits. The hypomethylating activity of azacitidine and decitabine has demonstrated significant clinical efficacy in myelodysplastic syndromes (MDS) and acute myeloid leukemia (AML). In the phase III AZA-001 trial (NCT00071799), azacitidine significantly prolonged overall survival in higher-risk MDS compared with conventional care regimens (median 24.5 vs 15.0 months; hazard ratio 0.58, 95% CI 0.43–0.77; *P* < 0.001), while also improving complete and partial remission rates and delaying AML transformation [24, 25]. Similarly, a multicenter phase III study in older patients with newly diagnosed AML showed that decitabine achieved higher response rates and a trend toward improved overall survival compared with low-dose cytarabine or supportive care (7.7 vs 5.0 months in an unplanned survival analysis; hazard ratio 0.82) [26, 27]. Beyond these approved indications, azacitidine and decitabine are actively being evaluated in additional clinical trials across various hematologic and solid malignancies. Azacitidine has been tested in prostate cancer (NCT00384839) and, in combination with erlotinib, in advanced solid tumors (NCT00996515) to assess safety, tolerability, and preliminary anti-tumor efficacy. Decitabine has been studied in combination with ruxolitinib in patients with accelerated or blast-phase myeloproliferative neoplasms (MPN) or post-MPN AML (NCT02076191) to determine optimal dosing, safety, and potential therapeutic benefit. Collectively, these studies highlight the broader translational potential of nucleoside analog DNMTis, and additional trials are ongoing to further explore their safety and efficacy in cancer therapy.

#### Non-nucleoside DNMTis

Non-nucleoside DNMTis have gained interest due to their DNA-incorporation-independent mechanisms, allowing inhibition of DNMTs in both dividing and non-dividing cells, thus expanding their therapeutic potential. One of the most studied is RG108, a small molecule that directly binds the catalytic pocket of DNMT1, leading to reversible, non-cytotoxic demethylation in various preclinical cancer and inflammatory models [28]. SGI-1027, another early non-nucleoside inhibitor, targets multiple DNMT isoforms and has shown promising in vitro activity, although further development has been limited [29]. Recent efforts have focused on improving selectivity and expanding therapeutic synergy. For instance, GSK3685032 is a highly selective DNMT1 inhibitor currently under investigation for its immunomodulatory effects, including upregulation of PD-L1, enhancement of T cell infiltration, and sensitization to immune checkpoint blockade. Similarly, MC3343, a novel DNMT1 inhibitor, has demonstrated potent reactivation of tumor suppressor genes and is under evaluation for solid tumor applications. Another emerging agent, CM-272, is a dual inhibitor targeting DNMT1 and G9a, offering a synergistic epigenetic blockade and demonstrating robust anti-tumor activity in hematologic malignancies and immune-responsive solid tumors [30].

#### Natural compounds-derived DNMTis

In addition to synthetic compounds, a growing number of natural products have demonstrated DNMT-inhibitory activity, attracting considerable interest due to their low toxicity, availability from dietary sources, and multifaceted biological effects. These compounds often exhibit pleiotropic mechanisms, targeting not only DNA methylation but also pathways involved in oxidative stress, inflammation, cell cycle regulation, and apoptosis, making them appealing candidates for cancer chemoprevention or adjuvant therapy.

Curcumin, a natural polyphenol antioxidant extracted from the rhizome of turmeric, has been proven to inhibit the proliferation of various cancer cells and induce apoptosis. However, the specific mechanism by which it induces DNA demethylation and the association between DNA demethylation and apoptosis remain unclear. Ma et al. used human gastric cancer cells (hGCCs) as a model to explore the mechanism of action of curcumin and found that high concentrations of curcumin increased intracellular ROS levels through pro-oxidative effects, causing mitochondrial damage and DNA damage [31]. On the one hand, curcumin inhibits the proliferation of hGCCs in a dose-time-dependent manner and induces apoptosis. On the other hand, down-regulation of DNMT1 leads to global DNA demethylation during cellular DNA replication, and it has been confirmed that this demethylation is a downstream result of the activation of the DNA Damage Response (DDR) pathway. This study provides a molecular mechanism basis for curcumin in the treatment of gastric cancer related to epigenetic regulation. In another study, researchers took three different subtypes of breast cancer cell lines as the subjects and explored the role of curcumin through various experimental techniques. They found that it could biaxially regulate the expression of BRCA1 and SNCG genes by regulating DNA promoter methylation (restoring the expression of highly methylated BRCA1 and inhibiting the expression of hypomethylated SNCG). Moreover, it can inhibit cell proliferation, indicating that curcumin may serve as a potential treatment option for triple-negative breast cancer (TNBC) and cancer prevention for BRCA1 methylation carriers [32]. Clinical exploration has also made positive progress. A Phase I clinical trial involving 15 patients with advanced colorectal cancer showed that oral curcumin remained well-tolerated for up to 4 months without dose-limiting toxicity. When the dose is 3.6 g/day, curcumin and its metabolites can be detected in plasma and urine, and they can effectively inhibit LPS-induced prostaglandin E₂ (PGE₂) production [33].

Epigallocatechin gallate (EGCG), the most abundant polyphenolic compound in green tea, has also been confirmed to be a potential epigenetic modulator. In breast cancer research, for MDA-MB-231 and MCF-7 cell lines, after EGCG treatment, the transcriptional levels of DNA methyltransferases (DNMT1, DNMT3a, DNMT3b) were significantly downregulated, and the degree of DNA methylation in the promoter region of the tumor suppressor gene SCUBE2 decreased simultaneously. The expression of this gene is reactivated, thereby inhibiting the growth, migration, and invasion abilities of breast cancer cells. When EGCG is used in combination with epigenetic inhibitors, such as sulforaphane, it can synergistically promote histone H3 acetylation and DNA demethylation, thereby enhancing the anti-tumor effect [34]. Another study found that EGCG can regulate the expression of tumor suppressor genes through a dual mechanism. On the one hand, it inhibits the activity of DNMT (especially DNMT3B) and HDAC; On the other hand, it directly binds to their active sites, ultimately reversing the hypermethylation status of the promoters of tumor suppressor genes RARβ, CDH1 and DAPK1, and reactivating these genes [35]. Recent clinical investigations have further highlighted the translational potential of EGCG. In NCT06924749, oxygen-nebulized EGCG is being evaluated for treating COVID-19–related pulmonary injury in cancer patients, building on its antiviral and radioprotective properties. Meanwhile, NCT05039983 explores an EGCG solution as supportive therapy to alleviate dysphagia in advanced esophageal cancer, focusing on safety, dosing, and preliminary efficacy. Together, these trials underscore EGCG’s expanding clinical relevance as a multifunctional adjunct for cancer-related complications.

Similarly, Quercetin is a flavonoid abundant in onions, apples, and berries. Recent experimental studies have provided clear evidence that quercetin exerts anti-tumor effects through direct epigenetic regulation. In leukemia cell lines, quercetin treatment markedly reduced the mRNA and protein levels of DNMT1 and DNMT3a, as well as class I histone deacetylases (HDAC1/2), leading to global DNA hypomethylation and promoter demethylation of pro-apoptotic genes such as BCL2L11 and DAPK1 [36]. In the xenograft mouse model, 120 mg/kg quercetin was administered every 4 days for 21 days. After the test, it was found that DNMT1 and HDAC1/2 in the tumor tissue were significantly downregulated, while the transcriptional levels of pro-apoptotic genes such as BAX, BNIP3, and APAF1 were significantly increased, and the tumor volume was significantly reduced [36]. Quercetin is increasingly being evaluated in clinical studies for its cancer-preventive potential. The ongoing trial NCT03476330 investigates whether quercetin can delay or prevent squamous cell carcinoma in patients with Fanconi anemia, highlighting its potential to reduce the need for aggressive oncologic treatments.

Other examples include genistein, a soy isoflavone, which has been reported in preclinical studies to inhibit DNMT and HDAC activity, reactivate ERα expression in breast cancer cells, and enhance tumor sensitivity to hormonal therapy [37]. Resveratrol has also been shown to exert indirect DNMT inhibition and epigenetic reactivation of silenced genes, while modulating SIRT1 and AMPK pathways [37, 38]. Besides, boswellic acids, sulforaphane (from cruciferous vegetables), and apigenin have also exhibited DNMT inhibition in preclinical models, although with varying potency and specificity [39]. These findings primarily highlight their mechanistic and chemopreventive potential.

Despite their relatively low binding affinity compared to synthetic DNMT inhibitors, these natural compounds offer a safer profile for long-term use, especially in pre-cancerous lesions, chronic inflammation-related cancers, or maintenance therapy. But they commonly face major translational bottlenecks, including poor solubility, low stability, and limited bioavailability. Ongoing research aims to improve their bioavailability and target specificity, including the development of nanoformulations or derivatives with enhanced pharmacokinetics [40]. For example, after intratumoral injection of EGCG-functionalized gold nanoparticles, the tumor volume significantly decreased within 28 days, and the effect was significantly better than that of free EGCG [41]. In the colorectal cancer model, the tumor growth inhibition rate of mice orally administered PH-sensitive EGCG-chitosan nanoparticles was also significantly higher than that of the free EGCG group [41]. Similarly, to break through delivery barriers, a variety of nanomedicines have emerged. In the triple-negative breast cancer model, the oral bioavailability of quercetin loaded polymer nanoparticles (PLGA-TPGS, Qu-NPs) was superior to that of free quercetin. The tumor suppression rate in 4T1 tumor-bearing mice was significantly improved, and lung metastasis was reduced [42]. The mechanism is related to the fact that Qu-NPs more effectively inhibit the proliferation and migration of cancer cells by down-regulating urokinase-type plasminogen activators (uPA). More advanced preparations have also achieved the combined delivery of quercetin and therapeutic nucleic acids, such as the co-delivery of quercetin and shTERT siRNA by RGD-modified mesopore silicon nanoparticles, which can induce apoptosis through the p53/Bax pathway and achieve better tumor suppression effects in ovarian cancer xenograft models [43]. Together, these findings indicate that natural compounds such as EGCG, curcumin, and quercetin function as weak but biologically relevant DNMTis, especially when combined with nanotechnology-based delivery systems to overcome poor bioavailability. While their standalone efficacy may be modest, they hold promise as adjuvants to synthetic epigenetic drugs or immunotherapies due to their low toxicity and multi-target regulatory capacity. In the future, continued advances in nanomaterial technology and clinical research are expected to make nanodelivery-based strategies for natural compounds a safer and more effective option for cancer therapy, thereby accelerating the translation of natural bioactive molecules from basic research to clinical application.

In recent years, combination therapies involving DNMTis and other epigenetic or immunomodulatory agents have gained traction. Synergistic interactions with HDAC inhibitors, BET inhibitors, immune checkpoint inhibitors, and DNA damage response agents have been reported in multiple preclinical and early-phase clinical studies, offering a multi-pronged approach to reverse epigenetic silencing, overcome drug resistance, and enhance anti-tumor immunity [29, 44]. Overall, the development of DNMTis has transitioned from broad-spectrum cytidine analogs to selective, isoform-targeted agents with improved pharmacokinetics and reduced toxicity. Ongoing research is also exploring their potential in non-oncologic diseases, such as autoimmune disorders, neurodegeneration (e.g., Alzheimer’s disease), and metabolic syndromes, suggesting a broader future impact of DNMT inhibition beyond cancer.

### Histone deacetylase inhibitors

**Fig. 2:**
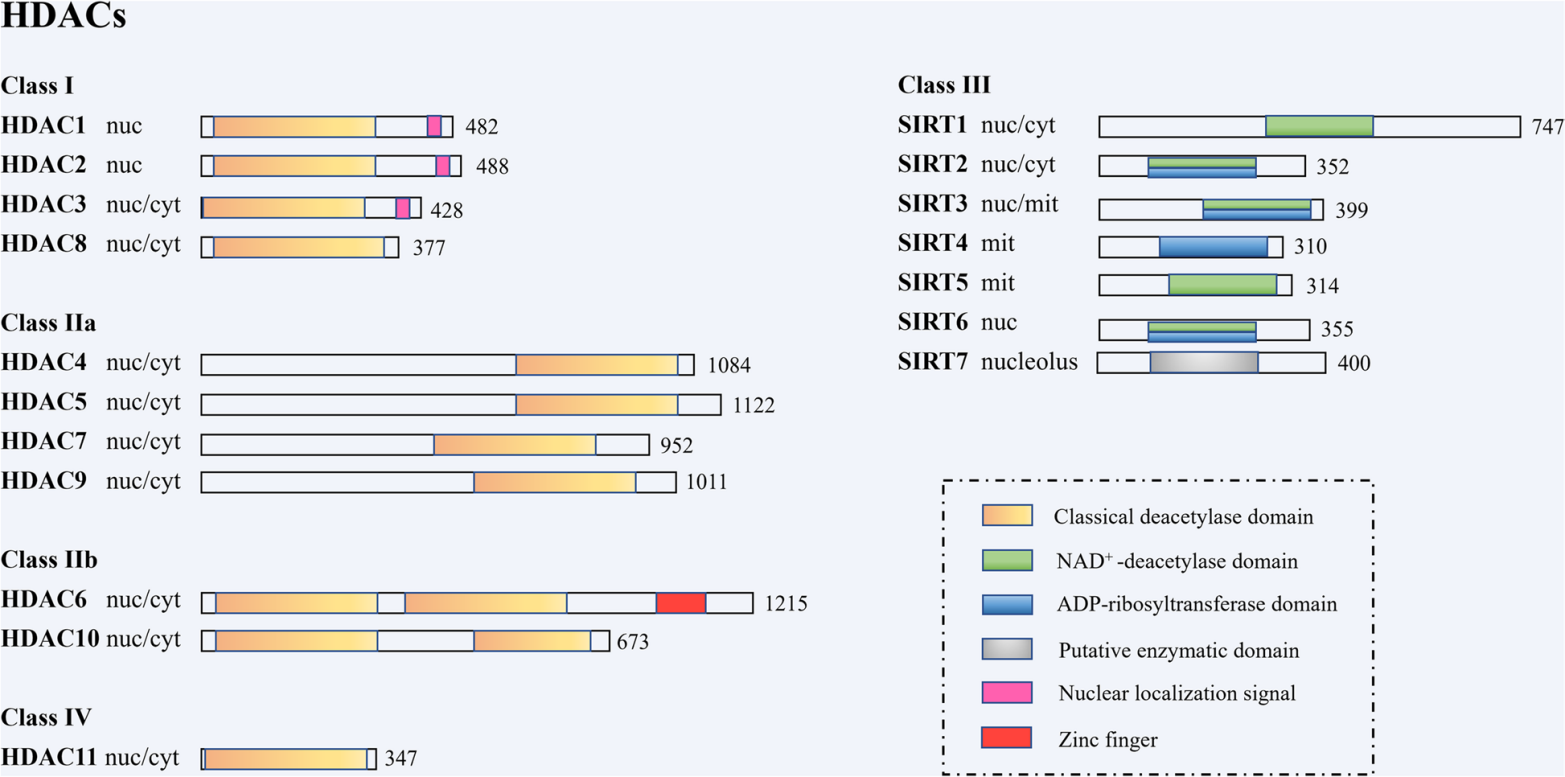
Domain organization of human HDACs. Human HDACs are classified into Class I (HDAC1-3, 8), Class IIa (HDAC4, 5, 7, 9), Class IIb (HDAC6, 10), Class IV (HDAC11), and Class III Sirtuins (SIRT1-7). The schematic illustrates the domain organization of human HDACs, with the total number of amino acid residues indicated on the right side of each protein. Only the longest isoform of each HDAC is presented, although many members exist in multiple isoforms. Enzymatic or putative enzymatic domains are highlighted in color to denote catalytic regions. Subcellular localizations are indicated as follows: nuc (nuclear), cyt (cytoplasmic), and mit (mitochondrial)

HDACs comprise a family of enzymes that regulate chromatin structure and gene expression by removing acetyl groups from lysine residues on histone and non-histone proteins, thereby promoting chromatin condensation and transcriptional repression [45]. Based on sequence homology and functional characteristics, HDACs are categorized into four classes: class I (HDAC1, 2, 3, 8), class II (further divided into IIa: HDAC4, 5, 7, 9, and IIb: HDAC6, 10), class III (Sirtuins: SIRT1-7), and class IV (HDAC11) [46]. The domain architecture serves as a foundation for understanding the structural and functional diversity among HDAC family members (Fig. 2).

Under normal physiological conditions, HDACs play a vital role in regulating gene expression, maintaining genome stability, and modulating cellular responses such as differentiation, proliferation, and apoptosis [47, 48]. However, abnormal HDAC expression contributes to tumorigenesis through silencing of tumor suppressor genes, disruption of cell cycle checkpoints, inhibition of apoptosis, promotion of metastasis, and modulation of the immune microenvironment [49, 50]. Class I HDACs, typically localized in the nucleus, are often overexpressed in colorectal, breast, prostate, and gastric cancers, where they drive uncontrolled proliferation and resistance to cell death [51]. Class II HDACs, which shuttle between the nucleus and cytoplasm, participate in tissue-specific gene regulation and influence processes such as epithelial-mesenchymal transition (EMT) and angiogenesis. For example, HDAC4 and HDAC5 are linked to metastasis and chemoresistance [52–54]. Class IIb member HDAC6, predominantly cytoplasmic, is critical for protein degradation, autophagy, and immune evasion via its effects on substrates like α-tubulin and HSP90 [55, 56]. Sirtuins (class III HDACs), though structurally distinct and NAD⁺-dependent, regulate cancer metabolism, oxidative stress responses, and DNA repair, with SIRT1 functioning in both oncogenic and tumor-suppressive contexts depending on cellular conditions [57, 58]. HDAC11, the sole member of class IV, plays an emerging role in immune regulation and tumor immunosuppression [59, 60]. As such, HDACis have emerged as promising epigenetic therapeutics in cancer treatment. Based on their chemical nature and sources, HDACis can be broadly categorized into small-molecule inhibitors, metal-based compounds, and natural product-derived inhibitors [61–64]. In addition to these chromatin level influences, HDAC inhibitors also regulate multiple intracellular signaling pathways, which are closely related to the survival, metabolism, and immune regulation of cancer cells. Among these the status of Tumor Protein 53 (p53) plays a decisive role. Existing studies have shown that HDAC inhibitors can stabilize p53 via acetylation. The mutation status of p53 directly affects the fate of tumor cells. Specifically, in wild-type p53 cells, this acetylation is more likely to induce apoptosis, while in mutant p53 cells, it may be more inclined towards autophagy, clearly demonstrating the regulatory role of p53 status on the efficacy of HDAC inhibitors [65]. Similarly, signal transducer and activator of transcription 3 (STAT3) serves as a "bridge molecule" between epigenetic regulation and oncogenic pathways, further connecting the action networks of the two types of inhibitors. Lee et al. found that acetylated STAT3 plays a key role in the methylation of tumor suppressor gene promoters. It can bind to DNMT1, promoting DNMT1-mediated methylation and silencing of tumor suppressor genes. Resveratrol can achieve demethylation and restore the expression of tumor suppressor genes by inhibiting the acetylation of STAT3 and destroying the STAT3-DNMT1 complex. This mechanism also shows therapeutic potential in various tumors. For instance, in triple-negative breast cancer, resveratrol can reactivate estrogen receptor α (ERα) and enhance tamoxifen sensitivity. In melanoma, it reveals the association between ERα methylation and tumor progression, providing a theoretical basis for cancer treatment targeting acetylated STAT3. [66].

#### Synthetic small-molecule HDAC inhibitors

Small-molecule HDAC inhibitors are a well-established class of epigenetic drugs designed to inhibit the catalytic activity of HDACs, leading to hyperacetylation of histones and non-histone proteins, chromatin decondensation, and reactivation of tumor suppressor genes [67, 68]. These inhibitors typically function by chelating the Zn^2^⁺ ion in the HDAC active site, blocking deacetylation. Several have received regulatory approval for cancer treatment. Vorinostat, the first FDA-approved HDAC inhibitor, is used to treat cutaneous T-cell lymphoma (CTCL), inducing apoptosis, cell cycle arrest, and differentiation [69]. Panobinostat, a pan-HDAC inhibitor, has been approved in combination with bortezomib and dexamethasone for relapsed or refractory multiple myeloma [70]. Romidepsin, a cyclic peptide prodrug, selectively inhibits class I HDACs and is also approved for CTCL and peripheral T-cell lymphoma (PTCL) [71]. Belinostat has demonstrated efficacy in PTCL with a favorable safety profile [72]. These agents have shown synergistic effects when combined with other therapies like proteasome inhibitors or immune checkpoint blockade. Ongoing trials are investigating HDACis in solid tumors such as glioblastoma, colorectal, and breast cancers. Additionally, subtype-selective HDACis such as HDAC6-targeting Citarinostat and Rocilinostat are under clinical evaluation, aiming to reduce off-target toxicity while enhancing tumor-specific activity.

Although first-generation HDACi such as vorinostat, panobinostat, and romidepsin demonstrated encouraging preclinical activity, their broad inhibition of multiple HDAC isoforms has resulted in a characteristic on-target, off-tumor dose-limiting toxicity (DLT) profile in the clinic. Because HDAC enzymes are essential for transcriptional and acetylation homeostasis in normal tissues, non-selective agents frequently induce hematologic adverse events (thrombocytopenia, neutropenia, anemia), gastrointestinal effects (nausea, vomiting, diarrhea), and, in some cases, cardiac complications that complicate dose escalation and chronic administration. Early-phase studies of vorinostat reported DLTs including grade 3 emesis and febrile neutropenia in combination regimens, alongside additional non-DLT hematologic events observed across schedules [73]. Overall, while HDAC inhibitors have validated HDACs as therapeutic targets, their broad isoform inhibition limits clinical application due to toxicity. Current efforts are focused on developing subtype-selective, better-tolerated HDAC inhibitors to improve efficacy and safety in both hematologic and solid malignancies.

#### Metal-based HDAC inhibitors

Metal-based HDAC inhibitors represent an emerging class of epigenetic therapeutics that utilize the coordination chemistry of metal ions to enhance potency, stability, and selectivity. As HDACs depend on a zinc ion in their catalytic core, many metal-based inhibitors are designed to interfere with Zn^2^⁺ coordination. For instance, copper(II) complexes bearing hydroxamate or Schiff-base ligands mimic classical HDAC pharmacophores while introducing redox activity, potentially generating reactive oxygen species (ROS) for enhanced anticancer effects [74]. Ru(II)-arene complexes, such as RAPTA-T, selectively inhibit HDACs and show antiproliferative activity in breast and prostate cancer models [75]. Ru(III) complexes like KP1019 function as prodrugs, activated by tumor-specific reduction environments, leading to selective cytotoxicity [76]. Gold(III)-based inhibitors, such as Au-dithiocarbamate and Au-NHC (N-heterocyclic carbene) derivatives, show promise in targeting HDAC6 and inducing apoptosis in cancer cells, including leukemia and glioblastoma. These metal complexes often possess dual functionality, such as HDAC inhibition and DNA interaction or ROS induction. Preclinical data suggest they are particularly effective in multidrug-resistant cancers, though concerns about biocompatibility and toxicity remain. Optimization of metal ligands and targeted delivery systems is currently a key focus to improve their translational potential.

#### Natural product-derived HDAC inhibitors

Natural compounds provide a rich source of HDAC inhibitors with unique scaffolds, high specificity, and lower toxicity [77]. Trichostatin A (TSA), a fungal metabolite, is one of the earliest discovered HDACis, potently inhibiting class I and II HDACs and widely used in epigenetic studies [77, 78]. Romidepsin, a natural cyclic peptide from Chromobacterium violaceum, is FDA-approved and functions by releasing a thiol group that chelates Zn^2^⁺ in the HDAC active site [79]. Largazole, a marine-derived thioester-containing depsipeptide, selectively targets class I HDACs and exhibits strong anti-proliferative activity in colon and pancreatic cancer models [80–83]. Other microbial HDACis such as Trapoxin A, Depudecin, Chlamydocin, and HC-toxin also inhibit HDACs with varied selectivity and mechanisms, including covalent binding to active sites or allosteric inhibition. These natural inhibitors serve as lead compounds for the synthesis of analogs with improved pharmacokinetic profiles. In preclinical cancer models, many of these compounds have demonstrated apoptosis induction, inhibition of angiogenesis, and sensitization to chemotherapy [84–87]. Their complex structures offer novel binding mechanisms that differ from classical zinc-chelators, and some can bypass resistance pathways. As such, they continue to be valuable in drug discovery and chemical biology research for next-generation HDAC-targeted therapies.

HDACis represent a diverse and rapidly evolving class of epigenetic therapeutics that have shown remarkable potential in cancer therapy. By targeting histone deacetylases, these agents modulate chromatin structure and gene expression, thereby reversing aberrant epigenetic silencing of tumor suppressor genes and restoring normal cellular functions such as cell cycle regulation, apoptosis, differentiation, and immune surveillance [52, 88]. Over the past two decades, multiple HDACis have entered preclinical and clinical development, with several already receiving FDA approval for the treatment of hematologic malignancies such as cutaneous T-cell lymphoma and multiple myeloma [89]. Despite these advances, challenges remain, particularly in improving isoform selectivity to minimize off-target effects and toxicity while enhancing therapeutic efficacy. Isoform-selective HDACis are emerging as a promising strategy to precisely modulate disease-relevant pathways with reduced systemic side effects, especially given the distinct biological roles of different HDAC isoforms in various cancers. Moreover, rational drug design, including the incorporation of targeted delivery systems, prodrug strategies, and combination regimens, is being actively explored to overcome resistance and enhance antitumor responses.

### Histone Demethylase Inhibitors (HDMis) and Histone Methyltransferase Inhibitors (HMTis)

Histone methylation is a key epigenetic modification involved in regulating gene expression, chromatin structure, and cellular behavior. Dysregulation of this process, particularly through aberrant activity of histone demethylases (HDMs) and histone methyltransferases (HMTs), is strongly associated with cancer progression. HDMs remove, while HMTs add, methyl groups to histone lysine residues, thereby modulating oncogene activation and tumor suppressor silencing [68, 69, 90–92]. Targeting these enzymes with small-molecule inhibitors (HDMis and HMTis) offers a promising strategy to restore normal epigenetic states, reactivate silenced genes, and enhance the sensitivity of tumors to existing therapies. Notably, several HDMs such as LSD1 (KDM1A) and JmjC domain-containing proteins (e.g., JMJD1A, JMJD2A) rely on cofactors like FAD or Fe^2^⁺ and oxygen to mediate oxidative demethylation, whereas LSD1 removes methyl groups from mono- and dimethylated H3K4 without requiring metal ions [73, 74, 90].

HMTs, particularly those containing SET domains, such as EZH2 and G9a, transfer methyl groups from SAM to histone lysines, influencing gene activation or repression in a context-dependent manner [76, 93–95]. Some non-SET-domain enzymes like DOT1L also play important roles by catalyzing H3K79 methylation. The specificity of HDMs and HMTs for particular lysine residues allows for precise epigenetic control, but their aberrant expression or mutation in cancers leads to pathological gene expression patterns [79–82, 96]. Thus, understanding the structural features and catalytic mechanisms of these enzymes has become pivotal for designing selective inhibitors as novel anticancer agents.

#### HDMis in cancer therapy

Overexpression or loss of regulation of HDMs has been implicated in the development of various cancers [97, 98]. By inhibiting HDMs, these drugs aim to reverse the silencing of tumor-suppressor genes or reprogram the cancer cell epigenome. Key HDM Inhibitors HDMis are typically small molecules that target the catalytic sites of specific HDMs. These inhibitors can be classified into several categories based on their chemical structures and the enzymes they target.

Peptoid-based inhibitors, such as GSK-J4, specifically target JMJD3 and UTX, two histone demethylases responsible for demethylating H3K27 [99, 100]. By inhibiting these enzymes, GSK-J4 can reactivate silenced tumor-suppressor genes, demonstrating significant anti-cancer activity in preclinical models [101]. Similarly, isoxazole-based inhibitors, such as GSK2879552, are designed to inhibit the KDM5 family of HDMs, particularly KDM5A and KDM5B, which are involved in the demethylation of H3K4 [102]. These inhibitors have shown promise in treating cancers where KDM5 overexpression contributes to tumor progression, including lung and prostate cancer. Furthermore, benzamide-based inhibitors, like INCB28060, specifically target LSD1 (KDM1A), an enzyme that demethylates H3K4me1/2 and H3K9me1/2 [103, 104]. Inhibition of LSD1 has been linked to reactivating silenced tumor-suppressor genes and inducing apoptosis, with notable anti-cancer effects observed in leukemia, breast, and prostate cancers [105–107]. LSD1 has recently been shown in multiple experimental studies to play a crucial role in tumor immune evasion. Genetic knockout or pharmacologic inhibition of LSD1 markedly enhances tumor immunogenicity by reactivating endogenous retroviral elements (ERVs) and triggering antiviral immune signaling. Sheng et al. confirmed through mouse melanoma and lung cancer models that the ablation or inhibition of LSD1 would significantly upregulate the transcription of ERVs in tumor cells, trigger the accumulation of double-stranded RNA (dsRNA), and subsequently activate type I interferon (IFN) signaling through the MDA5/MAVS pathway. Promote the infiltration of CD4⁺ and CD8⁺T cells into the tumor microenvironment, and at the same time reverse the resistance of B16 melanoma to anti-PD-1 treatment alone, enabling the combined treatment to significantly inhibit tumor growth and prolong the survival period of mice [108]. Similarly, Soldi et al. treated SWI/SNF-mutated ovarian cancer cells with the reversible LSD1 inhibitor SP-2577 (Seclidemstat) and observed enhanced ERV transcription, IFN pathway activation, and T-cell recruitment in 3D co-culture assays [109]. Furthermore, Hiatt et al. confirmed that pharmacologic LSD1 inhibition with Bomedemstat in small-cell lung cancer (SCLC) models promoted antigen presentation and sensitized tumors to immune checkpoint blockade [110]. Collectively, these experimental findings establish a direct mechanistic link between LSD1 inhibition and enhanced tumor immunogenicity, providing a solid biological rationale for combining LSD1 inhibitors with immune checkpoint inhibitors in future therapeutic strategies. Additionally, natural product-derived inhibitors, such as EGCG found in green tea, have demonstrated potential as HDM inhibitors, particularly against enzymes like JMJD2 [111]. Beyond inhibiting HDMs, these natural compounds also exert anti-cancer effects by modulating the epigenetic landscape and enhancing the efficacy of other cancer therapies. Together, these inhibitors represent a promising and diverse approach to epigenetic cancer therapy, offering potential therapeutic strategies for treating various malignancies.

#### HMTis in cancer therapy

HMTs catalyze the addition of methyl groups to histones, which often results in gene silencing [112]. The aberrant activity of these enzymes is strongly associated with the development and progression of various cancers, making them important therapeutic targets. Inhibiting HMTs can restore normal gene expression, reactivate tumor-suppressor genes, and suppress cancer cell growth. Several classes of HMT inhibitors have been developed, showing significant potential in cancer therapy.

Among these, SAM analogs are key players in HMT inhibition. EPZ-5676 is a potent and selective inhibitor of DOT1L, an HMT responsible for methylating H3K79, a modification commonly associated with cancers harboring MLL gene translocations, such as acute leukemia [113, 114]. EPZ-5676 disrupts the methylation-driven activation of oncogenes and has shown promising anti-leukemic effects in preclinical models, positioning it as a potential targeted therapy for MLL-rearranged leukemia. Another important class is pyrimidine-based inhibitors, such as UNC1999, which selectively inhibits EZH2, a member of the Polycomb Repressive Complex 2 (PRC2) responsible for methylating H3K27 [115, 116]. EZH2 is often overexpressed in various cancers, including lymphoma, prostate cancer, and breast cancer, and UNC1999 works by restoring the expression of tumor-suppressor genes, thus inhibiting cancer cell proliferation [117, 118]. It has demonstrated strong anti-cancer activity in both solid tumors and hematological malignancies in preclinical studies. Another promising EZH2 inhibitor, GSK343, also selectively inhibits H3K27 methylation and has shown efficacy in reactivating silenced tumor-suppressor genes, particularly in cancers with EZH2 mutations or overexpression. GSK343 has entered early-stage clinical trials, showing promise in lymphoma and prostate cancer treatment [119–121].

Recent findings have revealed that the PRC2/EZH2 complex, in addition to its well-established canonical function in chromatin repression, further exerts a regulatory effect on tumor immunity. PRC2-mediated histone methylation represents a widespread mechanism of immune evasion through suppression of MHC Class I antigen presentation in cancer. In a genome-wide CRISPR/Cas9 screen, Burr et al. demonstrated that genetic or pharmacologic inhibition of PRC2 restored MHC-I expression and reinstated CD8⁺ T-cell-mediated tumor control in vivo [122]. In head-and-neck squamous cell carcinoma (HNSCC), GSK126 or EPZ-6438 (tazemetostat) reduced H3K27me3 occupancy at the B2M promoter, upregulated MHC-I enhanced antigen-specific CD8⁺ T-cell proliferation/IFN-γ, and sensitized anti-PD-1-resistant tumors to checkpoint blockade in mice [123]. In addition, in lung squamous cell carcinoma, EZH2 inhibition (GSK126 or EPZ-6438) increased the MHCI/II mRNA and protein levels in 2D cell lines and patient-derived neoplasms (containing IFN-γ), remodeled chromatin markers at key sites, and improved the control of anti-PD-1 tumors in both autologous and homologous models [124]. Collectively, these preclinical investigations delineate a unified mechanism whereby PRC2-driven histone methylation serves as a key epigenetic suppressor of MHC-I expression across diverse tumor types. Highlighting a promising therapeutic avenue for integrating EZH2 inhibitors into immune-checkpoint-based cancer therapy.

Selective inhibition of EZH2 has been clinically validated by tazemetostat. In a phase II single-arm study of patients with advanced epithelioid sarcoma and loss of INI1 (NCT02601950), tazemetostat 800 mg twice daily achieved a confirmed overall response rate of 15% (95% CI 7–26%), including complete responses in ~ 2% of patients [125]. It demonstrates durable disease control in this ultra-rare sarcoma. In relapsed/refractory follicular lymphoma, a global phase II trial (NCT01897571) showed higher objective response rates in the EZH2-mutant cohort (69%, 95% CI 53–82%) than in the wild-type cohort (35%, 95% CI 23–49%), with durable remissions and manageable toxicity, leading to accelerated regulatory approval [126]. These phase II data provide level 2 evidence that EZH2-directed HMT inhibition can translate into durable clinical benefit in selected genetically defined lymphomas and sarcomas.

In addition to these, small-molecule inhibitors targeting other HMTs like CPI-1205 have emerged. CPI-1205 is a selective inhibitor of both EZH1 and EZH2, enzymes responsible for H3K27 methylation. By inhibiting these enzymes, CPI-1205 disrupts the transcriptional silencing of tumor-suppressor genes and inhibits the growth of EZH2-dependent cancers, including lymphoma and prostate cancer [127, 128]. Currently undergoing clinical trials, CPI-1205 is being tested in combination with other cancer treatments. Furthermore, natural product-derived inhibitors have garnered attention for their potential to target HMTs. Curcumin, a compound derived from turmeric, is well-known for its anti-cancer properties, and recent studies have shown that it can inhibit various HMTs, including EZH2 [129, 130]. This inhibition helps modulate the epigenetic landscape, potentially offering therapeutic benefits in cancers like breast, colorectal, and lung cancer. Additionally, EGCG is a key compound found in green tea, has shown potential not only as an HDM inhibitor but also as an HMT inhibitor [131–134]. By interfering with the activity of HMTs such as G9a and EZH2, EGCG reduces histone methylation and helps reactivate silenced tumor-suppressor genes, providing another valuable avenue for cancer therapy [135, 136].

Together, HDMis and HMTis, ranging from small molecules to natural products, represent a diverse and promising approach to modulating the epigenetic landscape in cancer, offering new potential strategies for therapeutic intervention. HDMis aim to reverse the aberrant demethylation of histones, while HMTis inhibit the addition of methyl groups to histones, both of which can restore normal gene expression and suppress cancer progression. Although these inhibitors are in the early stages of clinical development, their ability to target specific epigenetic alterations in cancer cells offers a promising strategy for precision cancer therapy. Ongoing research and clinical trials will continue to elucidate the full potential of HDMis and HMTis as cancer therapies and may lead to their integration into combination treatment regimens.

### Metabolic epigenetic modulators: IDH1/2 inhibitors and next-generation drugs

Mutations in isocitrate dehydrogenase 1 (IDH1) and IDH2 not only rewire cellular metabolic pathways but also reshape epigenetic landscapes to promote tumorigenesis, acting as key drivers in various cancers. Under physiological conditions, IDH1 (cytoplasmic) and IDH2 (mitochondrial) are NADP⁺-dependent enzymes that catalyze the oxidative decarboxylation of isocitrate to α-ketoglutarate (α-KG), generating NADPH in the process, which is essential for maintaining intracellular redox homeostasis. Cancer-related hotspot mutations, however, disrupt this normal enzymatic function. IDH1 mutations typically occur at the R132 site, while IDH2 mutations cluster at R140 and R172. These mutations confer a neomorphic activity, shifting substrate specificity from isocitrate to α-KG, thereby catalyzing the abnormal production of the oncometabolite D-2-hydroxyglutarate (2-HG) from α-KG. The accumulation of 2-HG competitively inhibits α-KG-dependent dioxygenases, including TET family DNA demethylases and Jumonji-C domain-containing histone demethylases. This inhibition results in widespread DNA hypermethylation and the aberrant accumulation of repressive histone marks (H3K9me and H3K27me), ultimately arresting cell differentiation and maintaining cells in an undifferentiated, pro-tumor state [137–139].

At the clinical level, this metabolism-epigenetic oncogenic axis has been successfully targeted by selective IDH inhibitors. Both the IDH1 inhibitor Ivosidenib and the IDH2 inhibitor Enasidenib can restore differentiation in IDH-mutated AML cells, and their clinical efficacy led to FDA approval for these genetically defined AML subtypes [137, 140]. As summarized in Table 3, additional IDH1/2 inhibitors, including next-generation dual inhibitors such as Vorasidenib and HMPL-306, are currently being evaluated in hematologic malignancies and solid tumors, highlighting the expanding clinical landscape of metabolic epigenetic therapy. In recent years, Vorasidenib, a dual IDH1/2 inhibitor with high blood–brain barrier penetration, has further expanded the application potential of IDH-targeted therapy in solid tumors. Vorasidenib significantly prolongs progression-free survival (PFS) in patients with grade 2 glioma carrying IDH mutations: the median PFS of the control group was 11.1 months, whereas that of the Vorasidenib group was extended to 27.7 months, with a hazard ratio (HR) of 0.39 [141]. This result not only confirms its potency in central nervous system (CNS) tumors but also sets a regulatory precedent for metabolic epigenetic therapy in solid tumors.

**Table 3:** Clinical Studies of IDH1/2 Inhibitors and Next-Generation Drugs

Drugs	Target	Indications	Key Clinical Outcomes	NCT identifier
Ivosidenib (AG-120)	IDH1	AML/MDS/solid tumors	Reduces 2-HG, restores differentiation	NCT02074839 (Phase 1, Recruiting), NCT02073994 (Phase 1, Completed)
Enasidenib (AG-221)	IDH2	AML/relapsed AML	Inhibits mutant IDH2, promotes differentiation	NCT01915498 (Phase 2, Completed)
Vorasidenib (AG-881)	IDH1/2 (dual)	Low-grade glioma (LGG) and other solid tumors	Prolonged PFS; CNS-penetrant dual inhibitor	NCT02481154 (Phase 1, Completed)
Olutasidenib (FT-2102)	IDH1	Relapsed/refractory IDH1-mutant AML, MDS, glioma	ORR in AML; reduces 2-HG	NCT02719574 (Phase 2, Completed)
BAY‑1,436,032	IDH1	IDH1-mutant tumors	Reduces 2-HG, reverses oncogenic metabolism	NCT02746081 (Phase 1, Completed)
HMPL-306	IDH1/2 (dual)	Advanced hematological malignancies with mIDH; Healthy volunteers (mass balance/PK study)	Plasma 2-HG reduction > 90%, DCR 100%, CNS penetration; PK and safety profile in healthy subjects	NCT04764474 (Phase 1, Terminated), NCT06671873 (Phase 1, Completed)

To further optimize the exposure of drugs in the central nervous system and expand the coverage of IDH mutations, a new generation of dual IDH inhibitors has entered the clinical trial stage. Rodon Ahnert and his team released the Phase I clinical trial data of HMPL-306 (Ranosidenib), which targeted patients with IDH-mutated gliomas and other solid tumors in Western populations [142]. Data show that HMPL-306 still maintains controllable safety even at the maximum test dose (400 mg once daily). In evaluable patients with low-grade glioma, this inhibitor reduced plasma 2-HG levels (a direct marker of IDH inhibition) by more than 90%, achieved a disease control rate of 100%, had a median PFS of approximately 20.5 months, and no dose-limiting toxicity was observed. Pharmacokinetic analysis confirmed that the drug exposure was dose-dependent and it had a strong ability to penetrate the blood–brain barrier, indicating that it could achieve sustained action on metabolic targets both throughout the body and in the central nervous system. Multiple Phase Ib/II clinical trials are currently underway to evaluate HMPL-306 in combination with chemoradiotherapy for glioma, AML, and cholangiocarcinoma. These studies aim to enhance the anti-tumor efficacy of HMPL-306 beyond its monotherapy activity through multimodal treatment strategies.

In conclusion, these studies have revealed a key paradigm in the field of precision oncology. Metabolic epigenetic reprogramming achieved through IDH inhibition can reverse carcinogenic epigenetic dysregulation, restore normal cell differentiation, and reshape the tumor epigenome. By targeting the specific metabolic vulnerability of IDH-mutated cancers, this strategy provides a unique intervention path for precise epigenetic therapy and opens new directions for the treatment of various IDH-driven malignant tumors.

### Bromodomain and Extra-Terminal Domain (BET) inhibitors

BET proteins are a subfamily of epigenetic reader proteins that recognize acetylated lysine residues on histone tails, thereby linking histone acetylation to transcriptional activation. This family comprises BRD2, BRD3, BRD4, and BRDT, all of which share two conserved bromodomains (BD1 and BD2) and an extra-terminal domain responsible for recruiting transcriptional coactivators and chromatin remodelers [143]. Among them, BRD4 is the most extensively studied, known for its ability to recruit positive transcription elongation factor b (P-TEFb), promote RNA polymerase II-driven elongation, and regulate gene expression even during mitosis [144, 145]. Through these mechanisms, BET proteins play crucial roles in cell cycle regulation, development, immune responses, and inflammation.

Importantly, aberrant BET activity, particularly that of BRD4, has been linked to the overexpression of oncogenes such as MYC, BCL-2, and NF-κB, which drive tumor proliferation, survival, and immune evasion [146, 147]. Consequently, BET proteins have emerged as attractive therapeutic targets in cancer and inflammatory diseases. Both small-molecule inhibitors and PROTAC-based degraders have been developed to block BET function or promote its degradation, respectively, as summarized in Table 4. [148, 149]. As research advances, BET proteins continue to represent a promising frontier in epigenetic therapy, especially in the context of transcriptional addiction in malignancies and dysregulated inflammatory pathways.

**Table 4:** Comparison Between Small-Molecule BET Inhibitors and PROTAC-Based BET Degraders

Category	Drug (NCT identifier)	Mechanism of Action	Biological Effect	Therapeutic Indications	Limitations/Challenges
Small-Molecule BET Inhibitor	JQ1	Competitive binding to BET BD1/BD2 domains	Disrupts chromatin binding; suppresses MYC/NF-κB	AML, TNBC, prostate cancer, inflammatory diseases	Short half-life; systemic toxicity
	I-BET762 (NCT01943851, NCT02706535, NCT01587703, etc.)	Competitive binding to BD1/BD2; blocks transcription factor recruitment	Suppresses oncogenic transcription programs	AML, TNBC, prostate cancer	Requires continuous dosing; reversible effect
	ABBV-075 (NCT04480086)	Inhibits BET bromodomains	Downregulates MYC and NF-κB signaling	AML, solid tumors	Dose-limiting toxicity; short half-life
	CPI-0610 (NCT02158858, NCT01949883, NCT02157636, etc.)	Blocks BET BD1/BD2 binding	Inhibits oncogene transcription	AML, myelofibrosis	Off-target effects; transient suppression
PROTAC-Based BET Degrader	dBET1	E3 ligase recruitment for BET degradation	Sustained BET protein degradation	DLBCL, multiple myeloma, TNBC	Clinical safety under investigation
	ARV-825	E3 ligase-mediated BET degradation	Sustained transcriptional inhibition; restores T cell function	Hematologic malignancies, TNBC	Off-target degradation risk
	MZ1	Targets BET proteins to proteasome	Sustained suppression of BET-dependent transcription	Preclinical hematologic and solid tumor models	Limited in vivo data; formulation challenges

#### Small-molecule BET inhibitors

The first generation of small-molecule BET inhibitors functions by competitively binding to the bromodomains (BD1 and BD2) of BET proteins, thereby displacing them from chromatin and preventing the transcriptional activation of target genes [150, 151]. These inhibitors have shown limited preclinical and clinical efficacy in hematologic malignancies, solid tumors, and inflammatory diseases [152]. First-generation BET inhibitors, including birabresib (OTX015/MK-8628), I-BET762, ABBV-075, BMS-986158, and RO6870810 have demonstrated only limited single-agent efficacy in early-phase clinical trials. Objective responses have been uncommon outside NUT carcinoma, and clinical benefit is frequently constrained by dose-limiting hematologic toxicities, particularly thrombocytopenia, which restricts sustained exposure and dosing intensity [153–156].

JQ1, a prototypical BET inhibitor, was the first-in-class compound developed to disrupt BRD4-chromatin interactions, leading to MYC downregulation and tumor growth inhibition [150, 157]. It has shown preclinical antitumor activity across AML, multiple myeloma, and solid tumor models through BET/BRD4 blockade and MYC-axis suppression. However, JQ1 itself is a research tool compound that has not entered clinical trials [158–162]. I-BET762, a pan-BET inhibitor originally developed by GlaxoSmithKline, has been discontinued from the oncology pipeline. All ongoing clinical trials have been withdrawn or terminated and discontinued from the oncology pipeline [163]. Although I-BET762 has been discontinued from oncology development, preclinical studies indicate it may still modulate NF-κB signaling, suggesting potential utility in autoimmune diseases [147, 164]. ABBV-075 (NCT04480086), developed by AbbVie, is not yet in the active clinical trial stage. Its Phase I pan-cancer trial has been completed, and its trial focusing on bone marrow fibrosis has been terminated [165]. CPI-0610, a BD2-selective BET inhibitor, has shown promising results in myelofibrosis, where it enhances the efficacy of JAK inhibitors by modulating inflammatory and fibrotic signaling pathways [166, 167]. The BD2-selective inhibitor ABBV-744 (AbbVie) showed robust preclinical efficacy in AR-positive prostate cancer and AML models, displacing BRD4 from AR-containing super-enhancers, inhibiting AR-dependent transcription, and reducing tumor growth in xenografts. Notably, ABBV-744 exhibited less thrombocytopenia/GI liability than the earlier dual-BD inhibitor ABBV-075 [168]. Moreover, recent mechanistic studies using GSK’s BD1- and BD2-selective chemical probes (e.g., iBET-BD1 and iBET-BD2) revealed that steady-state oncogenic transcription is largely BD1-driven, whereas inflammatory gene induction upon stimulation requires both BD1 and BD2 activity. These findings indicate that BD1-selective inhibition preferentially modulates cancer cell proliferation, while BD2 inhibition may more strongly regulate immuno-inflammatory responses, providing a rationale for indication-tailored BET targeting strategies in cancer and immune disorders [169].These findings indicate that BET inhibitors do not merely hold theoretical potential but have demonstrated preclinical efficacy in enhancing the effectiveness of immune checkpoint inhibitors, providing a strong rationale for translational development of such combinations in clinical settings.

While small-molecule BET inhibitors have demonstrated potent anti-tumor and anti-inflammatory effects, their clinical development has been hampered by dose-limiting toxicities, including thrombocytopenia and systemic toxicity. These limitations have driven the search for more reversible and targeted BET degradation strategies.

#### PROTAC-based BET inhibitors

Proteolysis-targeting chimeras (PROTACs) have emerged as a novel therapeutic strategy to overcome the limitations of traditional BET inhibitors [170, 171]. Unlike competitive inhibitors that merely block BET protein activity, PROTACs function by inducing the proteasomal degradation of BET proteins, leading to a more sustained and complete suppression of BET-dependent transcription.

dBET1, a first-generation PROTAC BET degrader, induces selective degradation of BRD4, resulting in robust MYC suppression and apoptosis in leukemia models [172, 173]. Compared to traditional BET inhibitors, dBET1 exhibits enhanced potency and prolonged therapeutic effects. ARV-825, another highly potent PROTAC BET degrader, has been shown to effectively target BRD4 in diffuse large B-cell lymphoma (DLBCL), multiple myeloma, and prostate cancer models [174, 175]. Preclinical studies indicate that ARV-825 is more effective in inhibiting tumor growth than conventional BET inhibitors. MZ1, a selective BRD4 degrader, has demonstrated superior efficacy in triple-negative breast cancer and leukemia models, suggesting a potential role in overcoming resistance to small-molecule BET inhibitors [150, 176, 177].

The future development of BET inhibitors focuses on enhancing the selectivity, reducing systemic toxicity, and improving pharmacokinetic profiles. For example, the development of bromodomain-selective BET inhibitors can achieve selective targeting of BD1 or BD2 to minimize off-target effects and reduce toxicity [150, 166]. In addition, synergistic effects can be achieved by combining BET inhibition with other epigenetic modulators, such as HDAC or DNMT inhibitors [178, 179]. Furthermore, it is also valuable to investigate the role of BET inhibition in enhancing anti-tumor immunity, particularly in combination with immune checkpoint inhibitors (ICIs) such as anti-PD-1/PD-L1 antibodies [180, 181]. In conclusion, BET inhibitors represent a powerful class of epigenetic therapies in cancer, inflammation, and autoimmune diseases. The integration of PROTAC technology and next-generation BET modulators holds promise for overcoming current limitations and expanding their clinical utility.

### Emerging epigenetic regulators

Although traditional epigenetic drugs, including DNA methyltransferase inhibitors, histone deacetylase inhibitors, histone methylation regulators, isocitrate dehydrogenase inhibitors, and BET inhibitors, have made progress in clinical practice, some key regulatory pathways remain unexplored. In recent years, some emerging epigenetic therapies have entered first-in-human trials (FIH) or received regulatory approval, gradually filling these gaps. P300 and CBP, as HATs, regulate the transcriptional activation of oncogenes like MYC and NF-κB [182]. Small-molecule inhibitors targeting P300/CBP have entered clinical development, with representative agents including CPI-637 (Phase I, for lymphoma) and A-485 (Phase I/II, for solid tumors and hematologic malignancies) [183]. These inhibitors exert anti-tumor effects by blocking HAT activity and suppressing oncogenic transcription, and preclinical studies have shown synergistic efficacy when combined with PD-1 inhibitors via enhanced antigen presentation [184, 185]. SMARCA2 is a key component of the SWI/SNF chromatin remodeling complex, and its dysregulation is associated with SMARCA4-deficient cancers. ACBI1 (ARV-771), a PROTAC-based SMARCA2 degrader, is currently in Phase I clinical trials for SMARCA4-deficient non-small cell lung cancer [186]. By inducing selective degradation of SMARCA2, ACBI1 restores normal chromatin remodeling and inhibits tumor cell proliferation [187]. Menin inhibitors target the Menin-KMT2A interaction, which is critical for leukemogenesis in KMT2A-rearranged acute leukemia [188]. Revumenib (approved by the FDA in 2024) is the first-in-class Menin inhibitor indicated for relapsed/refractory KMT2A-rearranged acute leukemia [189]. In addition to inhibiting leukemic cell proliferation, Revumenib enhances CD8 + T cell infiltration by upregulating tumor-associated antigens [190]. Protein arginine methyltransferase 5 (PRMT5) mediates symmetric dimethylation of histone H4R3, promoting oncogenic transcription. GSK3326595, a PRMT5 inhibitor in Phase II clinical trials, shows efficacy in non-Hodgkin lymphoma and non-small cell lung cancer. Preclinical studies indicate that GSK3326595 enhances the efficacy of PD-1 inhibitors by reducing Treg infiltration in the tumor microenvironment [191].

### Epigenetic modulation technologies: CRISPR-dCas9-based systems

In recent years, programmable epigenome editing using CRISPR-dCas9 platforms has emerged as a powerful alternative to traditional small-molecule epigenetic inhibitors. By fusing nuclease-deficient Cas9 (dCas9) to catalytic domains of epigenetic enzymes, researchers can modulate DNA methylation, histone acetylation, and chromatin accessibility at specific genomic loci with unprecedented precision. Unlike conventional DNMT or HDAC inhibitors that act globally, these systems enable locus-specific epigenetic regulation and minimize off-target effects. The application of this technology in tumor research has demonstrated clear therapeutic potential. Zahraei et al. designed the CRISPR-dCas9-TET1 system targeting the promoter region of miR-200c. This system can precisely target the miR-200c promoter and achieve local demethylation, successfully reactivating the expression of miR-200c. The upregulation of miR-200c is further achieved by down-regulating ZEB1, ZEB2 and KRAS, and simultaneously up-regulating e-cadherin. It reversed the EMT phenotype of tumor cells, thereby markedly reducing their invasiveness and proliferative capacity, demonstrating the therapeutic potential of targeted demethylation in cancer treatment [173]. In hepatocellular carcinoma (HCC), silencing of tumor suppressor genes such as HHIP, MT1M, PZP, and TTC36 is a key driver of carcinogenesis. Researchers employed the CRISPR activation system to precisely restore the expression of these silenced tumor suppressor genes. Reactivation of these genes directly inhibits the proliferation and migration of liver cancer cells, providing strong evidence that site-specific gene activation can counteract oncogenic programs [192]. In addition to regulatory efficacy, delivery efficiency and persistence of action are the core challenges restricting the transformation of epigenomic editing technology. Recently, Xu et al. developed an epigenome-reactivating virus-like particle (eVLP)-based instantaneous delivery system capable of efficiently delivering the dCas9-TET1 epigenetic editing complex. This platform effectively reactivated genes previously silenced by CRISPRoff and sustained their expression for several days, representing a significant advancement toward overcoming the long-standing challenges of limited delivery efficiency and transient activity in epigenome editing [193].

## Immune modulation by epigenetic drugs

Epigenetic drugs not only directly inhibit tumor growth through transcriptional regulation, but also profoundly reshape the tumor immune microenvironment. More and more evidence indicate that chromatin modifiers can induce dual reprogramming of tumor cells and immune cells, transforming the "immune desert" microenvironment into a highly immunogenic and therapeutic response state. A growing number of studies have demonstrated that epigenetic mechanisms tightly regulate key immune-related molecules, including cytokines, chemokines, transcription factors, and immune checkpoint receptors, at both the DNA and histone levels. For instance, modifications such as DNA methylation, histone acetylation, and methylation at promoter or enhancer regions control the expression of immune effector molecules (e.g., IFN-γ, Granzyme B, IL-12), immune suppressive mediators (e.g., IDO1, TNF-α), and checkpoint receptors (e.g., PD-1, CTLA-4, TIM-3). These findings are summarized in Table 5. To clarify the mechanism of this multi-dimensional interaction, this section will analyze three interrelated dimensions of immune regulation, particularly how epigenetic therapy enhances tumor immunogenicity, regulates immune checkpoints and co-stimulatory signals, and reprograms immune cell function. In conclusion, these dimensions form a coherent mechanism framework that closely links epigenetic remodeling with anti-tumor immunity.

**Table 5:** Epigenetic Regulation of Immune Molecules and Pathways in the Tumor Microenvironment

Molecule	Type/Function	Cell Type(s)	Epigenetic Regulation	Immunological Function
Granzyme B	Cytotoxic effector protease	CD8⁺ T cells	Increased H3K9Ac at promoter following TCR stimulation	Enhances cytotoxic T-cell activity and tumor cell killing
IFN-γ	Th1-type cytokine, pro-inflammatory	Naïve & memory CD8⁺ T cells	Promoter DNA demethylation in memory cells enables expression	Sustains memory T-cell responsiveness and anti-tumor immunity
IL-2	T cell growth factor	Activated CD8⁺ T cells	Activation-induced promoter demethylation maintained in memory phase	Supports clonal expansion and long-term T-cell persistence
IL-12	APC-derived cytokine promoting Th1 polarization	DCs, macrophages, T & NK cells	↑ H3K4me3, ↓ H3K27me2 at p35/p40 promoters	Amplifies Th1-type responses and IFN-γ production
TNF-α	Pro-inflammatory cytokine	Macrophages, T cells	Promoter DNA methylation represses expression within tumor microenvironment	Epigenetic silencing contributes to immunosuppression in tumors
CXCL9	T cell–recruiting chemokine	DCs, macrophages	Promoter hypermethylation inhibits transcription	Reduced T-cell infiltration and impaired immune surveillance
IDO1	Immune suppressive enzyme	Tumor cells, DCs	Promoter demethylation increases expression	Enhances immune escape
FoxP3	Treg transcription factor	Regulatory T cells	CpG methylation at enhancer region controls Treg lineage commitment	Stable FoxP3 expression maintains suppressive Treg phenotype
Batf3	cDC1 transcription factor	DCs	Promoter demethylation required for lineage specification	Ensures proper antigen cross-presentation to CD8⁺ T cells
STING	Cytosolic DNA sensor	Tumor cells	Promoter hypermethylation silences cGAS/STING; reversed by DNMTis	Reactivation restores innate sensing and type I IFN signaling
PD-1/CTLA-4	Immune checkpoint receptors	DCs, macrophages, T cells	Combined DNA and histone methylation repress transcription	Contributes to T cell exhaustion and immune evasion
MHC-I/MHC-II	Antigen presentation molecules	Tumor cells, DCs	Histone acetylation and DNA hypomethylation promote expression	Enhances antigen presentation and T-cell activation
ARG1	Immunosuppressive metabolic enzyme	Myeloid-derived suppressor cells	Promoter hypomethylation upregulates ARG1	Promotes local immunosuppression by depleting L-arginine
STAT1/STAT3	Signal transducers of cytokine signaling	T cells, macrophages	Histone acetylation/methylation balance regulates transcription	Alters inflammatory vs. suppressive phenotypes in immune cells

### Enhancing tumor immunogenicity (“Cold” → “Hot”)

The key to transforming immunosuppressive "cold tumors" into immunoinvasive "hot tumors" lies in enhancing the immunogenicity of the tumor. By activating antigen presentation, recruiting effector T cells, and breaking immune escape, a suitable microenvironment for immune checkpoint blockade (ICB) therapy is created. Epigenetic therapy, which precisely regulates gene expression, has become a key means to achieve this transformation. Through multidimensional interventions, such as DNA methylation and histone modification, it has constructed a comprehensive "immunogenicity enhancement network".

DNMTi triggers a "virus-simulated response" by silencing endogenous reverse transcription elements (ERVs). Abnormal transcription of ERV generates double-stranded RNA, which can be recognized by the intracellular MDA5/MAVS pathway and thereby activate the type I interferon (IFNα/β) signaling pathway. The activation of this pathway can not only up-regulate the expression of molecules related to antigen processing and presentation (e.g., MHC-I and TAP transporters), but also induce the secretion of chemokines to recruit CD8 + T cells, promoting the enhancement of tumor immunogenicity from both the "antigen presentation" and "immune cell recruitment" links [194, 195]. In addition to DNA methylation regulation, histone modification-targeted epigenetic interventions can also modulate immunogenicity, and targeted inhibition can effectively reverse immune evasion. The Polycomb Repressive Complex 2 (PRC2), a key histone modification complex, has EZH2 as its catalytic subunit, which can silence genes related to the MHC-I antigen presentation pathway by catalyzing H3K27me3 modification, helping tumors achieve immune escape. Inhibiting PRC2 can restore the expression of these genes, rebuild the antigen-presenting ability of tumor cells, and enable tumors to be recognized by cytotoxic T cells [122]. Meanwhile, specific HDACis further enhance "tumor visibility" by regulating the immune proteasome and MHC molecules. This type of inhibitor can selectively up-regulate the expression of the core components of the immunoproteasome (LMP2/PSMB9), optimize the processing efficiency of tumor antigen peptides, and simultaneously increase the expression level of MHC-I/II molecules on the surface of tumor cells, making it easier for cytotoxic T cells to recognize tumor antigens. This dual enhancement of "antigen processing—presentation" not only directly improves the killing efficiency of T cells, but also has a synergistic effect with anti-PD-1 antibodies, significantly increasing the response rate of ICB treatment [196]. More broadly, research on epigenetic therapy in non-small cell lung cancer (NSCLC) models has further revealed the deep logic of its regulation of immune responses. Epigenetic therapy links MYC attenuation with the reversal of immune evasion and the transformation of T-cell status to a memory/effector phenotype, further promoting the ICB response [197].

### Modulating immune checkpoints and co-stimulatory molecules

Epigenetic drugs can differentially regulate the expression of immune checkpoint molecules and the characteristics of the tumor microenvironment (TME) by targeting distinct epigenetic regulators, thereby creating conditions favorable for immune checkpoint inhibitor (ICI) therapy. HDAC inhibitors, through epigenetic reprogramming, not only significantly upregulate the expression of PD-L1/PD-L2 on tumor cells and myeloid cells but also promote the conversion of the TME toward an inflammatory phenotype. This dual effect, upregulation of PD-L1/PD-L2 and enhancement of inflammatory TME characteristics, can sensitize tumors in various contexts, such as melanoma and non-small cell lung cancer, rendering them more responsive to PD-1/PD-L1 blockade therapy [198, 199].

Unlike HDAC inhibitors, BET bromodomain inhibitors suppress PD-L1 gene transcription via a BRD4-dependent mechanism while simultaneously alleviating T cell exhaustion. This dual effect of reducing PD-L1 expression and restoring T cell function produces a significant synergistic effect with anti-PD-1 therapy in vivo, further enhancing the anti-tumor immune response [180, 181]. From the perspective of clinical transformation, the above-mentioned mechanism has been preliminarily verified. In Phase I/II studies targeting advanced solid tumors, the combination of the DNA methyltransferase inhibitor Aza and pembrolizumab demonstrated good feasibility and immune activity, providing direct support for the clinical value of the epigenetic drug-ICI combination strategy [200]. In conclusion, although HDAC inhibitors and BET inhibitors regulate immune checkpoints through different pathways, both can enhance the efficacy of ICI by reshaping the TME or repairing T cell function. The clinical data of azacitidine combined with pembrolizumab further confirm the translational potential of this epigenetic-ICI combination regimen, laying the foundation for the subsequent development of precise combination therapy.

### Modulating immune cell function

Epigenetic drugs not only exert anti-tumor effects by regulating the epigenetic state of tumor cells but also act directly on immune cells, ranging from optimizing T cell function to reshaping the phenotype of myeloid cells. These actions collectively dismantle immunosuppressive barriers and enhance anti-tumor immunity.

At the T-cell level, various epigenetic drugs precisely remodel T-cell function by targeting distinct epigenetic regulators. For instance, LSD1 inhibitors can significantly improve the differentiation quality and effector capacity of CD8⁺ T cells, enhancing their in vivo persistence in adoptive T-cell therapy (ACT) while maintaining an activated state that strengthens responsiveness to PD-1 blockade [201, 202]. Likewise, BET inhibitors play a pivotal role in repairing T-cell exhaustion in the microenvironment of chronic diseases. In chronic lymphocytic leukemia (CLL), BET inhibition restores T-cell proliferation and effector programs by reversing multi-receptor depletion, thus offering an important approach to overcome T-cell dysfunction under chronic inflammatory conditions [203].

In addition to regulating T cells, the reprogramming of myeloid cells by epigenetic drugs contributes substantially to remodeling the tumor immune microenvironment. Low-dose HDAC inhibitors, for instance, can reprogram tumor-associated macrophages (TAMs) toward an M1-like, pro-inflammatory phenotype with anti-tumor activity, while simultaneously reducing the recruitment of myeloid-derived suppressor cells (MDSCs) into tumors. This process not only enhances antigen presentation but also facilitates the initiation of T-cell activation [204, 205].

### Epigenetic regulation of oxidative stress and ROS-induced tumor cell death

Beyond acting through antigen presentation and immune cell remodeling, some epigenetic interventions can also directly induce intrinsic oxidative imbalance and cell death in tumors, and crosstalk with immunogenic cell death pathways. Emerging evidence indicates that epigenetic regulators not only regulate gene transcription but also induce the accumulation of ROS by altering cellular REDOX homeostasis, ultimately mediating tumor cell death. Several HDACis and DNMTis can enhance mitochondrial oxidative stress by disrupting antioxidant defense pathways and altering the expression of redox-regulating enzymes. A typical representative of this type of mechanism is the triple epigenetic inhibitor UVI5008, which can simultaneously inhibit histone deacetylases HDACs, DNMTs, and silent information regulators. In tumor models, UVI5008 not only activates the death receptor pathway but also significantly increases intracellular ROS levels, thereby inducing apoptosis. Importantly, this effect can still be maintained even in tumor cells with p53 mutations or TRAIL resistance, fully demonstrating that ROS generation is the core mediating factor of its cytotoxicity [206]. This discovery breaks the limitation that "epigenetic drugs rely solely on transcriptional regulation", revealing their ability to directly kill cancer cells through oxidative stress. More recently, epigenetic therapy has been shown to induce mitochondrial-mediated oxidative stress. Fresquet et al. demonstrated that treatment with hypomethylating agents reactivated endogenous retroelements, initiating a viral mimicry response that reprogrammed cancer cell metabolism away from glycolysis toward oxidative phosphorylation. This metabolic shift caused hyperactivation of the electron transport chain, excessive mitochondrial ROS accumulation, and ultimately caspase-independent cell death [207]. Collectively, these findings establish ROS induction as an important and complementary mechanism of action for epigenetic drugs. Recognition of this pathway not only strengthens the mechanistic foundation of epigenetic therapy but also provides a rationale for exploring combinatorial approaches with radiotherapy, ferroptosis inducers, or other redox-modulating agents to maximize therapeutic benefit.

## Therapeutic applications and clinical translation

At present, the therapeutic application and clinical translation of epigenetic modulators have entered a stage of rapid expansion. In recent years, a decisive shift from in vitro mechanism validation to in vivo efficacy and clinical concept validation has been witnessed, highlighting the true therapeutic potential of chromatin and DNA-targeted drugs. This section focuses on how to utilize these findings to reshape the modern oncology and immunotherapy paradigms. First, through molecular-defined single therapies, then through a reasonable combination strategy of epigenetic reprogramming and immune activation, and finally through examination to determine the translational considerations for clinical success, including biomarker design, drug resistance monitoring, safety management, and regulatory preparation. In conclusion, these advancements have transformed epigenetic regulation from an experimental concept into a clinically actionable discipline, linking basic biology with the interests of patients in the real world.

### Monotherapy applications

In the past years, the therapeutic landscape of epigenetic modulation has expanded from mechanistic exploration to tangible clinical benefit, particularly in genomically stratified malignancies. The most notable breakthrough is vorasidenib, a dual IDH1/2 inhibitor that achieved a landmark success in the phase III INDIGO trial, showing a clinically meaningful prolongation of progression-free survival (PFS) and delayed time to next intervention in patients with grade 2 IDH-mutant glioma [141]. This pivotal result led to its FDA approval in 2024, marking the first systemic therapy for this subset of patients. Similarly, the menin inhibitor revumenib, targeting KMT2A rearrangements and NPM1 mutations in acute leukemia, demonstrated deep and durable remissions in phase I/II studies [208], culminating in FDA approval in 2024 as the first agent in its class. These successes highlight a critical paradigm: precision-guided epigenetic therapies are most effective when molecular biomarkers tightly align with the underlying pathogenic circuitry. Beyond efficacy, optimization of treatment convenience also propels clinical adoption. The oral formulation of decitabine/cedazuridine has proven bioequivalent to intravenous dosing, enabling outpatient regimens that improve compliance and accessibility [209]. Collectively, these monotherapy advances signify the maturation of epigenetic therapy into an evidence-based precision modality, bridging mechanistic innovation and real-world deliverability.

### Combination with immunotherapy

Following the progress of monotherapy, combining epigenetic modulators with immune checkpoint blockade has become a compelling strategy to convert immunologically “cold” tumors into “hot” ones. Epigenetic drugs can reprogram antigen presentation pathways, bolster interferon signaling, and reverse T-cell exhaustion, thereby sensitizing tumors to immune attack. However, durable clinical efficacy remains challenging. For instance, the ECHO-206 Phase I/II trial, which evaluated the combination of azacitidine, epacadostat, and pembrolizumab, demonstrated acceptable safety but limited objective responses in heavily pretreated tumors, highlighting that the timing, sequencing, and tumor context of epigenetic priming are critical [200]. In terms of mechanism, EZH2 inhibition has now been shown to more directly enhance tumor immunogenicity. DuCote et al. demonstrated that EZH2 blockade upregulates both MHC-I and MHC-II machinery in lung squamous carcinoma, thereby improving antigen presentation and potentially enhancing T cell recognition [124]. Similarly, it has been reported that EZH2 inhibitors can reprogram the tumor microenvironment and enhance T-cell-mediated anti-tumor immunity, suggesting a translational synergy with immunotherapy [210]. More importantly, LSD1 inhibitors offer a complementary perspective. Activating CD8^+^ T cells with LSD1 inhibitors before adoptive transfer can enhance their in vivo persistence, reduce exhaustion, and improve anti-tumor effects, including when used in combination with PD-1 blockers [211]. Together, these studies underscore that the epigenetic-immune interface represents a promising frontier for precision immuno-oncology, provided that patient stratification, sequence design, and biomarker-driven selection are rigorously applied.

### Clinical translation

To achieve broad clinical benefits for epigenetic therapy and epigenetic-immunotherapy combination therapy, it is necessary to construct an integrated framework covering biomarker-driven stratification, treatment sequence design, drug resistance monitoring, safety management, and drug administration optimization. The implementation of this framework is not only an extension of the practical application of the concept of precision oncology but also a core support for promoting the efficient transformation of therapies from the laboratory to clinical practice.

First and foremost, precision screening at the molecular level remains the primary prerequisite for clinical benefits. Existing clinical evidence has clearly verified the matching value of the "biomarker-therapy" pairing. For instance, IDH-mutated gliomas exhibit selective responses to the IDH1/2 dual-target inhibitor Vorasidenib, while leukemia patients carrying KMT2A rearrangements or NPM1 mutations. Treatment with the Menin inhibitor Revumenib can lead to durable remission [141, 208, 212]. Cases with "clear targets and predictable responses" not only provide strict criteria for patient screening but also serve as important bases for regulatory authorities to approve indications. For example, during the approval process, the FDA explicitly designates molecular characteristics such as "IDH mutations" and "KMT2A rearrangements" as core indication labels for vorasidenib and revumenib [141, 208, 212]. Furthermore, the exploration of biomarkers for combination therapies is also advancing. For tumors with high EZH2/PRC2 complex activity or low activation of antigen presentation signaling pathways, the regimen of EZH2-targeted drugs combined with immune checkpoint inhibitors has a sound biological rationale. The value of these biomarkers lies in their ability to identify potential beneficiaries in advance, prevent mismatched patients from experiencing ineffective treatment and related toxicities, and provide precise guidance for the clinical positioning of combination therapies.

Second, the choice of treatment sequence has profound implications for the immune efficacy of combined epigenetic and immunotherapy. Inappropriate sequencing may directly weaken synergistic effects. Studies have explored the "epigenetic priming" strategy, which involves first administering epigenetic modulators (e.g., hypomethylating agents like azacitidine) followed by sequential immune checkpoint inhibitors (e.g., pembrolizumab), while also establishing and comparing regimens with concurrent administration of the two types of drugs. The results showed that pretreatment with azacitidine followed by sequential combination with immunotherapeutic agents only significantly reduced the density of FoxP3⁺ regulatory T cells in the tumor microenvironment, without effectively enhancing the infiltration of effector T cells (CD8⁺ T cells). When the two drugs were administered concurrently, there were no consistent changes in the number and ratio of CD8⁺ T cells and FoxP3⁺ T cells within the tumor, nor did they exhibit positive synergistic immune effects [200]. Thus, to accurately capture the optimal administration timing, it is necessary to introduce adaptive trial designs: by dynamically monitoring patients’ transcriptomic changes during treatment (e.g., peripheral blood single-cell sequencing, tumor tissue RNA sequencing) and integrating pathological and molecular information obtained from biopsies during treatment, real-time adjustments can be made to the administration sequence and dosage. This "real-time feedback and dynamic adjustment" model breaks the limitations of traditional fixed regimens and provides technical support for optimizing the immune synergy of combination therapies.

Third, with the widespread application of epigenetic therapy, acquired drug resistance has emerged as an urgent translational medicine issue to address. Recent structural biology and genomics studies have revealed the diversity of drug resistance mechanisms. For instance, in acute leukemia, secondary mutations in the MEN1 gene can be detected when patients experience relapse. These mutations are concentrated at the binding interface between the Menin protein and revumenib, and they alter protein conformation, leading to the loss of drug-binding ability and ultimately resulting in drug resistance [212]. This striking finding underscores the necessity of longitudinal molecular monitoring, which requires regular liquid biopsies or tumor tissue biopsies to dynamically track genetic variations and changes in protein expression during patients’ treatment, thereby enabling early warning of drug resistance. Meanwhile, it is also essential to concurrently develop adaptive therapeutic strategies that target drug resistance mechanisms.

Fourth, there is significant overlap in toxicity between epigenetic drugs and immunotherapies, which greatly increases the complexity of safety management. Epigenetic drugs (e.g., azacitidine) typically cause hematological toxicities, while immune checkpoint inhibitors (e.g., pembrolizumab) tend to trigger immune-related adverse events (irAEs). Their combination not only amplifies individual toxicity risks but may also increase potential synergistic harm, further elevating the toxicity burden. In conclusion, this toxicity overlap requires more targeted safety monitoring and close cooperation between oncology and immunology, which is key to balancing the therapeutic benefits of combination regimens and reducing avoidable harm to patients.

Finally, drug innovation and regulatory evolution are accelerating clinical translation. The development of oral decitabine/cedazuridine has made all-oral, dynamic regimens possible [213]. Its pharmacokinetic bioequivalence and intravenous administration have improved patient compliance and trial scalability. At the regulatory level, the FDA's approval of Vorasidenib and Revumenib further strengthens the approval logic of "biomarker-anchored indications", no longer relying solely on the traditional objective response rate (ORR). Instead, more precise endpoints such as "molecular remission" (e.g., clearance of IDH mutations and decrease in 2-HG levels) and "imaging remission" (e.g., prolonged progression-free survival of glioma) were incorporated into the assessment system. This innovative regulatory concept has opened a fast-track approval channel for more innovative therapies targeting epigenetic abnormalities. Overall, the construction and implementation of this integrated framework, from biomarker screening to treatment sequence optimization, from drug resistance monitoring to safety management, and then to the coordinated advancement of drug research and development and supervision, mark that epigenetic therapy has made a key leap from "conceptual innovation" to "clinically accessible precision therapy". In the future, with the integration of multi-omics technologies, artificial intelligence-assisted decision-making, and other tools, this framework will be further upgraded, bringing individualized clinical benefits to more cancer patients.

## Challenges and future perspectives

### Challenges

Epigenetic enzyme inhibitors have demonstrated significant therapeutic potential across various diseases [7, 214]. However, several critical challenges remain in their development and clinical translation. Addressing these obstacles will be crucial for advancing epigenetic-based therapies and improving patient outcomes.

#### Limited selectivity vs. on-target, off-tumor toxicity

The dose-limiting toxicity of first-generation epigenetic drugs, such as pan-HDAC inhibitors and nucleoside-based DNMT inhibitors, primarily stems from on-target, off-tumor effects rather than classical off-target toxicity. Because these agents indiscriminately inhibit essential epigenetic regulators required for transcriptional and differentiation homeostasis in normal tissues, particularly within the hematopoietic and immune systems, they significantly narrow the therapeutic index and limit clinical applicability [215]. Mechanistically, hematopoietic stem and progenitor cells (HSCs/HSPCs) depend on a tightly regulated balance of DNA methylation and histone deacetylation for lineage commitment and proliferation. Consequently, broad-spectrum inhibition of DNMTs or HDACs disrupts this equilibrium, leading to impaired hematopoietic differentiation and cytopenias, which clinically manifest as granulocytopenia, anemia, and thrombocytopenia, often accompanied by heightened susceptibility to infection [215, 216]. Supporting this, a large-scale pharmacovigilance analysis from the World Health Organization (WHO) VigiAccess database, comprising 23,763 adverse event reports for azacitidine and decitabine in myelodysplastic syndrome, identified hematologic and lymphatic toxicities, particularly bone marrow suppression, as the most common dose-limiting toxicities, followed by cardiovascular events. These findings confirm that the major clinical adverse effects result from non-selective “on-target” inhibition of DNA methylation, which affects not only malignant cells but also normal hematopoietic and cardiac tissues [217].

Therefore, overcoming this intrinsic limitation requires improving selectivity and delivery precision through multiple complementary strategies. At the molecular level, the design of isoform- or complex-selective inhibitors can minimize systemic perturbation. At the tissue level, targeted drug delivery approaches restrict drug exposure to vulnerable tissues like the bone marrow. At the mechanistic level, transient inhibition technologies such as PROTAC-mediated protein degradation can achieve temporary suppression of target proteins without continuous enzymatic blockade, thereby reducing residual functional interference and systemic toxicity.

#### Acquired and adaptive drug resistance

The reversible and dynamic nature of epigenetic modifications allows diseased cells to develop resistance to epigenetic inhibitors [218, 219]. Multiple resistance mechanisms have been identified, including compensatory activation of parallel epigenetic pathways, mutations of target enzymes, and upregulation of drug efflux transporters [220, 221]. For instance, after the action of BET inhibitors, BRD4 dissociates from chromatin. AML cells rapidly initiate "transcriptional reconstruction at the enhancer/super-enhancer level" through p300 (which can function in synergy with CBP); p300 is enriched in the enhancer/super-enhancer regions of oncogenes such as MYC, RUNX1, and CDK6, and maintains the H3K27ac level through acetyltransferase activity to rapidly restore the transcription of these genes from the initial down-regulated state, achieving "reconstruction of oncogenic transcriptional programs". With the evolution of drug resistance, this transcriptional state solidifies at the chromatin level (manifested as the stable expression of "evolutionary" drug resistance gene modules), forming a stable chronic drug resistance map [222]. Meanwhile, tumor cells will shift transcriptional dependence upwards to extension nodes (such as CDK9). Even if the initiation of transcription is suppressed, they can still maintain the expression of short-lived oncogenic proteins such as MYC and MCL1 through CDK9. This suggests that the network cascade inhibition of BET inhibitors and CDK9 inhibitors is expected to delay or even reverse such drug resistance. Another common mechanism is drug efflux. Upregulation of ABCB1/P-gp reduces intracellular effective exposure and induces cross-resistance. It is necessary to combine P-gp inhibition, substrate property, and structure optimization, or drug delivery engineering (nano/coupling) to counteract this [223]. In addition, the apparent pathway redundancy after DNMT inhibition (such as PRC2/H3K27me3 compensation) and the buffering effects of phenotypic plasticity and the immune microenvironment jointly weaken the drug efficacy, suggesting that synchronous design at both ends of the network node linkage and drug delivery pathways is the key to improving the sustained response rate [224].

#### Limited pharmacokinetics and bioavailability

Most epigenetic drugs currently face issues such as low oral bioavailability, short half-life, and uneven tissue distribution. Among these, drugs for central nervous system indications are particularly constrained by the blood–brain barrier (BBB), making the problem more pronounced. In the field of metabolic engineering, oral decitabine/cedazuridine (DEC/CED) achieves bioequivalence with intravenous decitabine area under the curve (AUC) by inhibiting cytidine deaminase (CDA), which has been verified in a randomized crossover design and subsequent follow-up, establishing a replicable paradigm of "enzymatic compatibility—oral administration". In terms of evidence for CNS drug accessibility, both preclinical studies and preliminary human imaging experiments on HDAC6 brain-imaging radioligands and compounds with brain-targeting selectivity have confirmed that: central target binding and pharmacodynamic detection are feasible. This breakthrough provides key tool support for the "CNS on-target-peripheral burden reduction" dose optimization strategy of epigenetic drugs [225, 226]. In terms of drug delivery engineering, nanoromidepsin, formulated as polymeric nanoparticles of romidepsin, has demonstrated prolonged circulation, increased tumor/blood ratio, enhanced efficacy, and improved tolerance in T-cell lymphoma models. It has shown potential for reengineering of "narrow-window HDACi" [227].

### Future perspectives

#### Domain/target selectivity and next-gen inhibitors

In the field of tumor epigenetic therapy, "precise targeting" has become the core direction to overcome the limitations of traditional drugs, as focusing on specific protein domains or family members allows it to retain anti-tumor activity while reducing off-target toxicity. This trend is particularly prominent in the development of targeted drugs for BET family proteins and EP300/CBP family HATs. In the field of BET protein targeting, although traditional pan-BET inhibitors can exert anti-tumor effects by inhibiting bromodomains, they are often accompanied by adverse reactions such as thrombocytopenia and gastrointestinal toxicity, which limit their clinical application. However, BD2-selective inhibitors (e.g., ABBV-744) can avoid this problem, as they retain anti-tumor activity while reducing the aforementioned toxicity [168]. This domain-selective therapeutic advantage stems from the functional differentiation between BD1 and BD2, and mechanistic studies have clarified their distinct roles in transcriptional regulation: BD1 is primarily responsible for maintaining homeostatic transcriptional processes, while BD2 regulates stress-responsive gene programs [169].

In addition to the precise regulation of bromodomains, inhibitors targeting the HAT family have also expanded new dimensions for epigenetic intervention, among which the value of EP300/CBP family HAT inhibitors is particularly prominent. Unlike traditional epigenetic drugs, these inhibitors can directly weaken the enhancer-related H3K27ac modification, a key marker of enhancer activity and oncogenic transcriptional addiction, thereby blocking the abnormal transcriptional programs of tumor cells from the source [228]. Their emergence not only enriches the tool library for precise epigenetic regulation but also provides new targets for the treatment of enhancer-driven cancers, such as some lymphomas and breast cancers. Comparative analysis shows that the transcriptional inhibitory efficacy of next-generation EP300/CBP compounds is superior to that of the first generation, laying the foundation for translational research on enhancer-driven cancer [229].

#### Targeted Protein Degradation (TPD/PROTACs)

Targeted protein degradation (TPD) technology, as a disruptive strategy in drug development in recent years, provides a brand-new approach for the intervention of difficult-to-drug targets by specifically eliminating pathogenic proteins rather than merely inhibiting their activity. Among them, PROTAC degraders of BET family proteins are typical application representatives of this technology. BET protein PROTAC degraders represented by ARV-771, ARV-825, and dBET series can efficiently eliminate BRD family proteins. Compared with traditional inhibitors, this type of degrader not only blocks the catalytic function of BRD proteins but also inhibits their role as signal scaffolds, thereby more thoroughly blocking downstream pathways and demonstrating stronger anti-tumor activity [230]. In-depth structural biology studies have further elucidated its mechanism of action: crystallographic analysis of the ternary complex formed by the PROTAC MZ1, VHL, and the BRD4 bromodomain 2 (BRD4BD2) revealed a synergistic protein–protein interaction interface. This discovery not only clarifies the selective mechanism and efficacy rules of degraders but also provides a structural basis for the rational design of TPD molecules [231]. In addition to BET proteins, TPD technology has developed a broader strategic system, including the "molecular glue" strategy mediated by phthalimide (CRBN) and the PROTAC strategy. The expansion of this technical concept has established a universal method for eliminating intracellular targets, completely revolutionizing the research and development paradigm of epigenetic drugs, shifting from the traditional "inhibitory function" to "protein clearance", providing a breakthrough solution for addressing the drug development challenges of epigenetic regulatory factors. [232]. In specific applications, TPD technology has demonstrated unique advantages in the treatment of tumors related to multi-comb inhibitory complex 2. The PROTAC degrader targeting EED not only degrades the EED protein but also synergically depletes EZH2 and SUZ12 in the PRC2 complex, resulting in a significant reduction in H3K27me3 levels and generating a potent anti-proliferative effect in PRC2-dependent tumor models [233]. EZH2-selective degraders (e.g., MS1943) complement the strategy of targeting EED by directly eliminating the EZH2 protein, while overcoming the application limitations of traditional EZH2 catalytic inhibitors (e.g., insufficient effect on mutant EZH2) [234].

In summary, the TPD technology centered on PROTAC has achieved in-depth intervention in disease-related pathways by precisely degrading specific epigenetic regulatory proteins. In the future, as TPD technology continues to mature, it is expected to open up new treatment approaches for more intractable diseases and promote a paradigm shift in drug development from "inhibition" to "degradation".

#### Dual-target strategies and drug delivery

In the field of precision oncology, dual-mechanism drugs have demonstrated unique therapeutic advantages by simultaneously acting on key pathways involved in tumor initiation and progression. CUDC-907 is a typical representative. As a dual-target inhibitor against HDAC and PI3K, it not only exerts a synergistic effect of chromatin remodeling and survival pathway inhibition but also exhibits significant monotherapeutic anti-tumor activity, offering a new direction for treating malignant tumors such as neuroblastoma (NB). Specifically, CUDC-907 functions via a dual mechanism. On the one hand, it targets Class I HDAC (primarily HDAC1/2/3). Inhibiting HDAC activity significantly increases the level of the histone acetylation marker H3K27ac in NB cells and xenograft tumor tissues, thereby inducing chromatin structure loosening and achieving chromatin remodeling. On the other hand, it reduces the level of phosphorylated AKT (p-AKT) by targeting the PI3K pathway, directly blocking the PI3K/AKT survival pathway. At the same time, it can also downregulate phosphorylated ERK (p-ERK) to inhibit the MAPK/ERK pathway, both of which are core pathway for NB cell proliferation and anti-apoptosis. Dual inhibition further impairs the survival capacity of tumor cells.

In terms of monotherapeutic activity, CUDC-907 exhibits remarkable efficacy. In vitro, it inhibited the proliferation of five NB cell lines in a concentration-dependent manner, with half-maximal inhibitory concentrations (IC₅₀) ranging from 5.53 to 46.22 nM. Moreover, CUDC-907 induced apoptosis, suppressed cell migration and invasion, and reduced stem cell-like properties of NB cells. Consistently, in vivo studies further validated its efficacy: oral administration of 25 mg/kg CUDC-907 significantly inhibited the growth of SK-N-BE(2) xenograft tumors without causing notable weight loss in mice, demonstrating a favorable balance between efficacy and safety [235]. Moreover, the dual HDAC-PI3K inhibitor CUDC-907 has significant single-agent activity in SCLC cells (inhibiting SCLC cell proliferation by downregulating MYC homolog, inducing G1 cell cycle arrest, promoting apoptosis, and impairing DNA double-strand break repair). Moreover, it has a synergistic effect with the PARP inhibitor olaparib and can enhance the anti-tumor efficacy in both SCLC cell lines and patient-derived xenograft (PDX) models, providing a reasonable combination treatment strategy for SCLC clinical research [236]. In addition to the development of dual-target inhibitors, the optimization of oral administration regimens is also an important direction in translational research on tumor treatment, aiming to enhance treatment convenience and patient compliance. For instance, the oral combination of decitabine and cedazuridine has been proven to achieve a drug exposure level comparable to that of intravenous administration and is more suitable for outpatients. At present, research on the combined treatment of this combination with drugs such as venetoclax is continuously advancing, and it is expected that the all-oral treatment plan will be extended to hematological tumors such as acute myeloid leukemia and myelodysplastic syndrome [209, 237]. In conclusion, whether it is targeted drugs like CUDC-907 that achieve multi-pathway synergistic inhibition through dual mechanisms, or oral decitabine/cedazuridine that optimize the administration route, they all provide more efficient and convenient new options for tumor treatment. They jointly promoted the development of tumor treatment towards precision, convenience, and low toxicity.

## Conclusion

Epigenetic enzyme inhibitors represent a powerful class of therapeutic agents with applications across a wide spectrum of diseases. To overcome current limitations, emerging strategies are being developed to enhance the specificity, efficacy, and durability of epigenetic therapies. For example, the emerging field of epigenetic transcriptomics, which focuses on RNA modifications has opened new avenues for therapeutic intervention [238]. RNA methyltransferase inhibitors, including METTL3 inhibitors, are being investigated for their role in cancer treatment. Targeting RNA modifications provides an alternative way of regulating gene expression without altering DNA or histone modifications, potentially leading to more reversible and tissue-specific effects. In addition, artificial intelligence and machine learning are increasingly being utilized for the design and optimization of epigenetic inhibitors. AI-driven drug discovery platforms enable the rapid screening of small molecules with high specificity, reducing the time and cost of developing novel epigenetic drugs. By leveraging cutting-edge drug design strategies, combination therapies, and personalized medicine approaches, the next generation of epigenetic therapies has the potential to revolutionize modern medicine.

## Data Availability

Not applicable.
